# Differential Demographic Trajectories and Polygenic Adaptation to Monsoon Seasonality in Sky Island Hercules Beetle Populations of the American Southwest

**DOI:** 10.1111/mec.70551

**Published:** 2026-09-25

**Authors:** Sean Chien, My‐Hanh Le, Heath Blackmon, Jen‐Pan Huang

**Affiliations:** ^1^ Texas A&M University College Station Texas USA; ^2^ Biodiversity Research Center, Academia Sinica Taipei Taiwan; ^3^ Ecology and Evolutionary Biology IDP Texas A&M University College Station Texas USA

**Keywords:** adaptation, conservation, demographic history, evolution, genomics, population genomics

## Abstract

Sky island mountain ranges of the American Southwest represent isolated habitat fragments surrounded by arid lowland barriers, making them powerful natural experiments for studying population divergence, demographic history and local adaptation. Here, we use whole‐genome resequencing of the Western Hercules beetle (*Dynastes grantii* Horn, 1870) across four primary lineages to characterize the genomic consequences of habitat fragmentation and climate‐driven isolation. Population structure analyses revealed strong hierarchical genetic differentiation consistent with isolation‐by‐distance, while ABBA‐BABA tests detected little to no evidence of post‐divergence gene flow among lineages. Coalescent modelling revealed synchronous demographic expansions during the Last Glacial Period followed by severe Holocene contractions across all lineages, with the Utah population experiencing the most pronounced bottleneck and maintaining persistently low effective population size (*N*_e_). The elevated *F*_ST_ but equivalent *D*_*XY*_ in Utah comparisons and elevated runs of homozygosity (ROH) indicate that its genetic distinctiveness reflects accelerated drift rather than ancient isolation. Genomic‐environment association analyses identified 1367 high‐confidence candidate loci associated with precipitation seasonality, with precipitation of the wettest quarter driving the strongest signal of polygenic adaptation. Monsoon moisture regimes are the primary selective axis shaping genomic divergence. Genetic offset projections identified Mt. Lemmon as the most vulnerable population under future climate scenarios, and genetic rescue donor analyses identified population‐specific sources for assisted gene flow. Our results demonstrate how integrating demographic inference, divergence genomics and landscape genomics with whole‐genome data, including historical museum specimens, can simultaneously advance understanding of evolutionary history and inform actionable conservation strategies in the future.

## 1 Introduction

A central goal of evolutionary biology is to understand how populations diverge in isolation, what drives adaptive differentiation across environmental gradients, and how gene flow influences the maintenance of genetic variation and adaptive potential in peripheral populations. Such information is critical not only in the face of rapid climatic changes that may impact the distribution and survival of endemic and endangered species (Love et al. 2023), but also to proactively predict future risks and opportunities for safeguarding biodiversity. The sky islands of the American Southwest represent a distinctive biogeographic system in which to address these questions. The isolated mountain ranges characterized by lush forest and seasonal monsoon are separated from each other by lowland desert basins, a landscape shaped by climate change over millennia. During cooler Pleistocene epochs, mesic forest habitat extended continuously across lower elevations (Betancourt et al. 1990), but as global temperatures warmed and the climate became increasingly arid, this once‐connected habitat fragmented and retreated upslope. Separated now by elevational gradients spanning over 1500 m, these mountains create a patchwork of cool, mesic forest habitat surrounded by arid lowlands that act as effective dispersal barriers for montane‐adapted species. This geographic fragmentation makes sky island systems uniquely powerful natural experiments for understanding fundamental evolutionary processes (Masta 2000; McCormack et al. 2008; Mitchell and Ober 2013; Smith and Farrell 2005).

While the southwestern sky islands have been a major focus of biogeographic research (Haire et al. 2022; Love et al. 2023; Frey 2026; Meyer et al. 2015; Moore et al. 2013), our understanding of population divergence and adaptation remains heavily skewed toward vertebrates. Previous studies on birds, mammals and reptiles have revealed intricate patterns of endemism and locally adapted populations across sky island systems (McCormack et al. 2008). Yet insects, which represent more than 99% of animal biomass and sustain the ecosystem functions that support these vertebrate communities, have received comparatively little genomic attention (Derkarabetian et al. 2016; Halbritter et al. 2019). Beetles and other arthropods offer unique advantages for studying how habitat fragmentation shapes populations. Their short generation times and ecological diversity allow evolutionary processes to unfold on observable timescales, making them ideal for detecting the genomic signatures of isolation and adaptation that would take generations to observe in longer‐lived vertebrates. Despite their ecological importance and evolutionary potential, detailed genomic characterization of how sky island fragmentation affects population structure, gene flow and adaptive divergence in these arthropods remains scarce.

**FIGURE 1:**
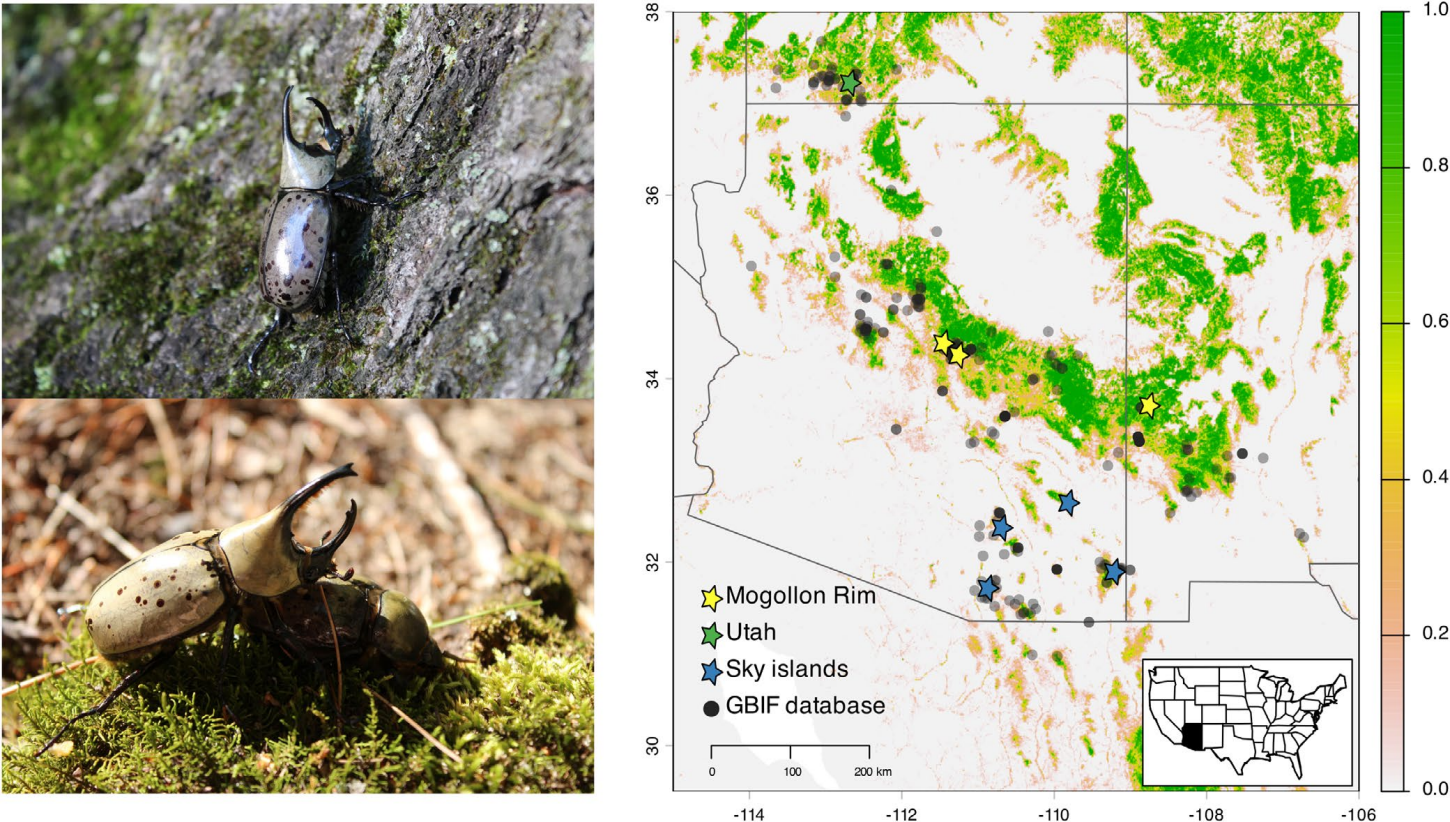
Tree cover and sampling locations for species distribution of *D. grantii*. Map showing tree cover across the southwestern United States with star symbols indicating the locations of sampled individuals. Species occurrence data were obtained from the Global Biodiversity Information Facility (GBIF; accessed April 23, 2024) (GBIF.org User 2026). Only samples from the United States are displayed. Samples from Mexico and those with unknown geographic origin are excluded from the map.

The Western Hercules beetle, *Dynastes grantii* Horn, 1870, is a charismatic species endemic to high‐elevation forest habitats of the American Southwest and northern Mexico (Morón 2009; Ratcliffe et al. 2013; Ratcliffe and Cave 2017) (Figure 1). Adult males, which can exceed 6 cm in length, have exaggerated horns that are used in male–male competition over resources such as mates and food (Menke and Parker 1988). Both males and females have white or greyish white body coloration with irregular black spots or markings that match the background coloration of their host plants (e.g., velvet ash). Moreover, the larval stage spans approximately 2 years and depends on large quantities of decaying wood for development, implying that their population size may be closely associated with the extent and health of forest ecosystems (McMonigle 2012). Although adults are winged and capable of flight, their large body size and heavy build might make them comparatively weak dispersers relative to smaller insects. That sustained movement across the arid, low‐elevation basins separating mesic montane forests is energetically costly. Adult dispersal across unsuitable lowland gaps exceeding 1500 m in elevation would be expected to reinforce isolation among mountain ranges. In addition to occurring throughout the sky island systems, the species is also distributed along the southern fringe of the Rocky Mountains, including populations in Colorado and Utah. Furthermore, its closely related species, *D. hyllus* , is broadly distributed across multiple ecoregions of the mountains of the Mexican Transition Zone (Morón 2009). Given that the genus has a South American origin (Dutrillaux and Dutrillaux 2013), colonization of western North America likely proceeded from south to north, suggesting that populations along the southern fringe of the Rocky Mountains in Colorado and Utah may represent more recent northward expansions.

In this study, we use the Western Hercules beetle as a model system to investigate how historical climate dynamics, habitat connectivity and projected future climatic change may shape the genetic diversity and evolvability of a charismatic species inhabiting the sky island system. We focus on four specific questions: (1) How is genetic variation structured across the sky island archipelago, and is there evidence of gene flow among lineages? (2) What demographic trajectories have these lineages followed, and do any populations show signatures of recent decline and inbreeding? (3) What environmental factors drive adaptive divergence? (4) How will projected climate change affect populations, and which are most vulnerable or best suited as donors for assisted gene flow? By integrating these analyses within a single whole‐genome dataset, we reconstruct the evolutionary history of a sky island insect while providing guidance for its conservation.

## 2 Methods and Materials

### 2.1 Sample Collection & DNA Isolation

We collected samples from various sky island mountain ranges in the American Southwest between 2023 and 2024, using a light‐sheet attraction method. Briefly, a 250 W mercury vapour was used to illuminate a white sheet at night, and attracted beetles were collected by hand directly from the sheet. These localities included Mt. Lemmon (Pima, AZ), Chiricahua Mt. (Portal, AZ), Mt. Graham (Graham County, AZ) and Mt. Wrighton (Santa Cruz County, AZ) (Figure 1). Upon collection, beetles were stored in 100% ethanol prior to DNA extraction. We supplemented our contemporary collections with historical specimens from Field Museum (Chicago, IL, USA), Museum of Southwestern Biology (University of New Mexico), Biodiversity Research Museum (Academia Sinica, Taiwan) and private sources to achieve broader geographic representation. These additional samples included individuals from Mogollon Rim (Payson, AZ and Reserve, NM); Cochise County, AZ; Kane County, Utah; and Bacanora, Sonora, Mexico (Table S1). A subset of samples from private collections with unknown locality data was also included.

Total genomic DNA was extracted from leg tissue using the DNeasy Blood & Tissue Kit (QIAGEN). To improve lysis of chitinous beetle tissue, legs were flash‐frozen in liquid nitrogen and homogenized to a fine powder prior to extraction, and the lysis step was extended to 18–24 h at 56°C to maximize DNA yield. The resulting extracts were submitted to Novogene (Sacramento, CA) or GENOMICS (New Taipei City, Taiwan) for library preparation and whole‐genome sequencing on the Illumina NovaSeq X Plus platform (PE150).

### 2.2 Variants Calling

Raw sequencing reads were aligned to the *D. grantii* reference genome (NCBI: GCA_029619325.2) using BWA‐MEM2 (v2.2.1) (Jung and Han 2022). Alignments were converted to BAM format with SAMtools (v1.15) (Li et al. 2009), sorted by genomic coordinate and filtered to retain only mapped reads with MAPQ ≥ 30. Duplicate reads were marked and removed using GATK MarkDuplicates (v4.5) (McKenna et al. 2010). Variant calling followed the GATK pipeline using GPU‐accelerated NVIDIA Parabricks (v4.5). Per‐sample variants were generated in gVCF mode with Parabricks HaplotypeCaller v4.5. Individual gVCFs were merged with GATK CombineGVCFs and jointly genotyped with Parabricks GenotypeGVCFs (v4.5) to produce a raw, multi‐sample variant callset. Filtering was performed in two steps using VCFtools (v0.1.16) (Danecek et al. 2011) through the whole analysis. To guide filtering thresholds, we first quantified sequencing depth and missing data at both the individual and site levels (empirical distributions provided in Figure S1). In the first step, we removed low‐quality individuals (MC1 and SR1) (mean depth < 5× and missingness > 30%) and retained only biallelic SNPs and removed indels. In the second step, additional filters (e.g., minor allele frequency (MAF), missingness/call rate and depth thresholds) were applied as needed for specific downstream analyses and are described in the corresponding sections. MAF thresholds were chosen to match the sensitivity of each analysis: a stringent cutoff for population structure (MAF ≥ 0.10, to restrict to common, informative variants), a permissive cutoff for demographic and divergence analyses (MAF ≥ 0.01, which rely on rare variants in the allele frequency spectrum), and an intermediate cutoff for genotype–environment association (MAF ≥ 0.05, standard for association testing to limit false positives from rare variants).

### 2.3 Population Structure and Gene Flow

For population structure and gene flow, we applied a common second‐stage filtering step in VCFtools (v0.1.16) to retain variants with MAF ≥ 0.10, site call rate ≥ 0.80, QUAL ≥ 20, mean site depth between 10× and 30×, and per‐genotype depth between 10× and 30×. Unless noted otherwise, all population structure results are based on this filtered SNP set.

To characterize the major axes of genetic variation, we performed Principal Component Analysis (PCA) using PLINK (v2.00a 3.7) (Purcell et al. 2007). To ensure that the analysis was not biassed by physical linkage between markers, we first performed linkage disequilibrium (LD) pruning. SNPs were pruned using a sliding window of 50 k variants, a step size of 10, and an *r* ^2^ threshold of 0.2. The resulting set of independent SNPs was then used to calculate the principal components. The final eigenvectors and eigenvalues were visualized in 2D and 3D representations of the genetic space. We estimated individual ancestry proportions and genetic clustering using ADMIXTURE (v1.3.0) (Alexander et al. 2009). To determine the most likely number of ancestral populations (*K*), we ran the software for *K* values ranging from 2 to 9. The optimal *K* value was identified by examining the cross‐validation (CV) error across all runs, where the *K* with the lowest error value represents the most predictive model. After identifying the best‐supported clustering levels, we visualized the genetic proportions to examine the hierarchical population structure and potential admixture across the population range.

We calculated *D*‐statistics (ABBA‐BABA tests), *Z*‐score and *f*_4_‐ratio using *Dsuite* (v0.5 r58) (Malinsky et al. 2021) to test for genomic signatures of gene flow among populations. The Utah population was specified as the outgroup for all comparisons based on PCA and ADMIXTURE results. We evaluated all possible topological combinations of the populations (*n* = 31, Unknown locality samples excluded (UN*)). Then, we excluded the historical sample (Cochise (C); 1960s) to test and distinguish contemporary gene flow patterns from signals driven by temporal divergence between historical and modern populations over approximately 60 years. We assessed statistical significance using a threshold of *p* < 0.05.

To evaluate the relationship between geographic and genetic distance (isolation by distance, IBD), we performed a Mantel test using a subset of individuals with confirmed locality data (*n* = 29, Unknown (UN*), Mexico (M), Cochise (C) samples were excluded). We first generated a genetic distance matrix in PLINK (v2.00a 3.7), utilizing the same LD‐pruning parameters as the PCA to ensure independent markers. Geographic distances were calculated from decimal coordinates using the Haversine formula (*distm* function, R package geosphere), which accounts for the curvature of the earth. The resulting genetic and geographic distance matrices were compared using a Mantel test with Pearson's correlation coefficient in the R package vegan (Dixon 2003). For localities lacking precise GPS coordinates (Payson, AZ and Reserve, AZ), we used representative coordinates for the named collection locality. Statistical significance was assessed using 9999 permutations with a fixed random seed (set.seed(4)) to ensure reproducibility.

**FIGURE 2:**
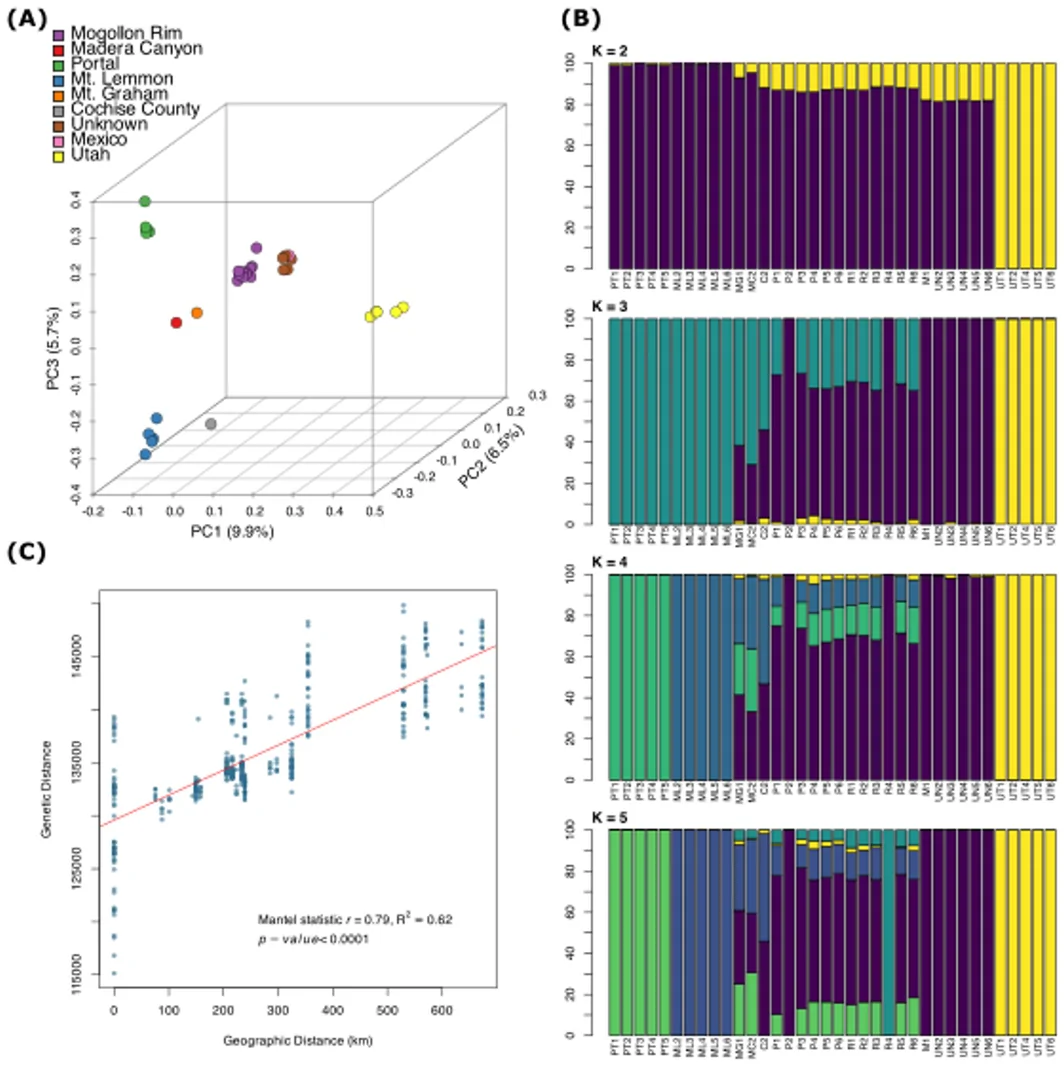
Population structure of *D. grantii* in the American Southwest. (A) Three‐dimensional projection of principal components analysis reveals hierarchical population separation across the southwestern sky island system. The Utah population is distinctly isolated along the positive PC1 axis, the Mogollon Rim clusters separately along the PC2 axis, while the southern sky island populations (Chiricahua Mt., Mt. Lemmon, Mt. Wrighton) form a loosely grouped cloud in the PC1–PC2 plane. Singletons from Mt. Graham and Mt. Wrighton occupies intermediate positions consistent with potential admixture or incomplete sampling of geographic intermediates. The Mexico sample and unknown‐origin individuals form a distinct cluster. (B) ADMIXTURE ancestry proportions. Stacked bar plots showing ancestry proportions inferred by ADMIXTURE for 38 *D. grantii* individuals at increasing numbers of ancestral populations (*K* = 2–5). Each vertical bar represents a single individual, with coloured segments indicating the proportion of ancestry from each inferred cluster (MC, Mt. Wrighton; MG, Mt. Graham; ML, Mt. Lemmon; PT, Chiricahua Mt.; C, Cochise CO., AZ; P, Payson, AZ; R, Reserve, AZ; M, Mexico; UN, Unknown; UT, Utah). (C) Isolation‐by‐distance (IBD) in *D. grantii* populations. Scatter plot showing the relationship between pairwise geographic distance (km) and genetic distance among 29 individuals in the data set (excluding unknown origin samples). A significant positive correlation (regression line) indicates that populations become genetically more differentiated as geographic distance increases.

Based on the initial assessment of population structure, we refined our dataset to focus on the four primary, well‐sampled lineages (Mogollon Rim (P* & R*, *n* = 12), Mt. Lemmon (ML*, *n* = 5), Chiricahua Mt. (PT*, *n* = 5) and Utah (UT*, *n* = 5)). Several groups represented by single individuals or lacking precise locality data (e.g., Mt. Wrighton (MC), Mt. Graham (MG), Cochise County (C) and Mexico (M)) were excluded from analyses requiring robust population‐level estimates. For all subsequent analyses, the first‐stage filtering consistently involved subsetting the VCF using VCFtools (v0.1.16) to include only these 27 individuals, The Mogollon Rim lineage was represented by two nearby sampling sites, Payson (*n* = 6) and Reserve (*n* = 6), which were treated as a single population based on their shared ancestry in the PCA and ADMIXTURE analyses (Figure 2). Any second‐stage filtering, such as specific thresholds for MAF, missingness or the inclusion of invariant sites, is detailed within the respective methodology sections for each analysis.

### 2.4 Demographic History and Coalescent Modelling

To reconstruct the historical changes in effective population size (*N*_e_), we used a sequentially Markovian coalescent approach using SMC++ (v1.15.4) (Terhorst et al. 2017). This method leverages both the site frequency spectrum (SFS) and linkage information from multiple individuals. For each of the four populations, we utilized the 11 largest anchored scaffolds to ensure sufficient genomic coverage for coalescent scaling. We first converted the filtered VCF into the SMC++ format using the *vcf2smc* command, treating each individual separately across the designated scaffolds. We then performed population‐specific *N*_e_ estimations using the *estimate* command and performed independent iterations per population to account for variation in the estimation process. Because SMC++ *N*_e_ and time estimates scale directly with the assumed mutation rate and generation time, both of which carry substantial uncertainty, we did not rely on a single point estimate. We re‐scaled the SMC++ trajectories across a range of mutation rates (3 × 10^−9^ and 5.8 × 10^−9^) (Huang 2019; Keightley et al. 2014; Miles et al. 2017; Xu et al. 2026) and generation times (1–2 years) (McMonigle 2012) to assess how these assumptions affect the absolute magnitude and timing of inferred *N*_e_ changes (Figure S3).

We infer recent demographic shifts (within the last 200 generations) using GONE2 (v2.0) (Santiago et al. 2025) to estimate contemporary *N*_e_. We filtered Stage 1 VCF for a MAF ≥ 0.01, a site call rate of 100%, and mean site/per‐genotype depth between 10× and 30×. To ensure robust LD estimates, the analysis was restricted to the largest anchored scaffolds. Input files were generated using PLINK (v2.00a 3.7). We ran GONE2 for each of the four lineages using a recombination rate of 2.48 cM/Mb (Wilfert et al. 2007). This approach allowed us to contrast the deep‐time demographic trends inferred by SMC++ with fine‐scale changes in *N*_e_ during the most recent evolutionary history.

We used δaδi (v2.4.3) (Gutenkunst et al. 2009) to evaluate two demographic scenarios from joint frequency spectrum (FS) to identify the most parsimonious history of divergence for each population pair. First, we used VCFtools (v0.1.16) to retain high‐quality sites by requiring a minimum quality score of 30, a site call rate ≥ 80% and a mean depth between 10× and 30×. To satisfy the assumption of independent observations required for FS analysis, we performed LD pruning using PLINK (v2.00a 3.7). SNPs were pruned using the same LD‐pruning parameters as the PCA. Following pruning, we generated the joint FS using *easySFS* (https://github.com/isaacovercast/easySFS). To maximize the number of segregating sites while accounting for missing data, we utilized the projection functionality in easySFS, down‐sampling each population to the optimal number of chromosomes based on a preview of the available SNPs.

Two demographic models were compared: (A) Strict Isolation (SI), assuming no post‐divergence gene flow; (B) Isolation with Size Change (ISC), which additionally allows each sub‐population to change size exponentially following the split. Migration was fixed to zero in both models. This assumption is supported by the absence of significant gene flow among the four lineages in ABBA‐BABA tests and the lack of admixed ancestry in ADMIXTURE analyses. The significant *D*‐statistic signals detected in the full dataset involved single‐individual localities (Mt. Graham, Mt. Wrighton and the historical Cochise sample) that were excluded from demographic modelling. Numerical integration was performed using a three‐level grid of points (pts = [*n, n* + 10, *n* + 20]) to ensure high‐accuracy extrapolation to the continuous limit. To ensure the identification of the global maximum likelihood and avoid local optima, we executed 100 independent replicates for each model starting from randomly perturbed initial parameters (*dadi.Misc.perturb_params*) and retained the replicate with the highest log‐likelihood. Models were compared using the Akaike Information Criterion (AIC = 2 k − 2lnL), where *k* is the number of free parameters.

### 2.5 Patterns of Genomic Divergence

We calculated pairwise *F*_ST_ (relative divergence) and *D*_*XY*_ (absolute divergence) to quantify genomic differentiation and diversity across the four primary lineages using pixy (v1.2.7) (Korunes and Samuk 2021). By comparing *F*_ST_ and *D*_*XY*_, we aimed to distinguish between recent sorting of ancestral polymorphisms and long‐term isolation among the lineages. To ensure accurate estimates of *D*_*XY*_, Stage 1 VCF file was filtered for a MAF ≥ 0.01, a site call rate ≥ 80%, a mean depth of coverage between 10× and 30×, and retained invariant sites. We calculated these statistics across the genome using a 5 kb sliding window approach. To test whether the high relative differentiation observed in the Utah population was driven by long‐term isolation or accelerated genetic drift, we compared the distributions of window‐based *F*_ST_ and *D*_*XY*_ values using Wilcoxon rank‐sum test and the difference in median values between population pairs to quantify the magnitude and direction of divergence. *p*‐values were adjusted (*p*.adjust, method = ‘BH’, R stats) for multiple testing using the false discovery rate (FDR) correction. Comparisons were considered significant if they showed both statistical support (FDR < 0.05) and exceeded a minimum effect size (absolute median difference > 0.01).

We performed runs of homozygosity (ROH) analysis for each population to understand patterns of genomic inbreeding and historical demography. We applied population‐specific variant filtering using VCFtools (v0.1.16). Stage 1 VCF file was filtered at a MAF ≥ 0.01, a site call rate ≥ 90%, a mean depth of coverage between 10× and 30×. ROH were identified using BCFtools *roh* (v1.19) and allele frequencies were estimated directly from each population‐specific VCF. Differences in the sum of ROH (S_ROH_), inbreeding coefficients (F_ROH_) and number of ROH segments (N_ROH_) among the four populations were assessed using the Kruskal–Wallis rank sum test (kruskal.test, R stats) and Post hoc pairwise comparisons were performed using the Wilcoxon rank sum test (wilcox.test, R stats) with Bonferroni correction for multiple testing.

### 2.6 Genomic Environmental Association (GEA)

To identify candidate loci involved in local adaptation across the Southwest, we performed two complementary GEA analyses: Redundancy Analysis (RDA) and Latent Factor Mixed Models (LFMM2). Prior to analysis, Stage 1 VCF file was filtered at a MAF ≥ 0.05, a minimum quality score of 20, and a site call rate ≥ 90% with a mean depth and individual genotype depth between 10× and 30×. The filtered VCF was converted to PLINK (v2.00a 3.7) format, with missing genotypes explicitly coded as ‘9’ to facilitate mean‐frequency matrix imputation in R. Environmental data were obtained from WorldClim 2 at a 30‐arcsecond resolution (Fick and Hijmans 2017). To minimize multicollinearity among the 19 bioclimatic variables, we applied a stepwise variance inflation factor (VIF) procedure (*vifstep*, usdm) (Naimi et al. 2014), which removes the variables iteratively with the highest VIF and recalculates until all remaining variables have VIF < 10. Our final models focused on three key precipitation variables: Precipitation of the Driest Quarter (PD), Precipitation of the Wettest Quarter (PW) and Precipitation of the Coldest Quarter (PC). These are the final predictor set and no further variable selection was performed. This combination of multi‐locus (RDA) and univariate (LFMM2) methods allowed us to identify both subtle polygenic shifts and large‐effect loci associated with regional moisture gradients.

For the RDA, we used the vegan package in R (Dixon 2003). We imputed missing genotypes using the mean allele frequency per locus and standardized environmental predictors. The significance of the full RDA model was assessed using 999 permutations (ANOVA‐like permutation test), and we identified outlier SNPs as those exceeding ±3 standard deviations (SD) from the mean loading on the first two RDA axes (Capblancq and Forester 2021; Forester et al. 2018). For the LFMM, we used the LFMM2 R package, which utilizes a ridge‐regression approach to account for unobserved population structure (Caye et al. 2019). Based on our previous PCA and ADMIXTURE results, we set the number of latent factors to *K* = 4. We ran separate models for each environmental predictor and calculated *p*‐values calibrated with the genomic inflation factor (GIF). To account for multiple testing, we applied a FDR threshold of 0.05. SNPs identified by both RDA and LFMM were categorized as high‐confidence concordant candidates for local adaptation.

To investigate the biological functions of the candidate adaptive loci, we performed a Gene Ontology (GO) enrichment analysis. We first annotated the *D. grantii* genome (NCBI: GCA_029619325.2) using BRAKER3 (Brůna et al. 2020, 2021; Buchfink et al. 2015; Gabriel et al. 2021; Gotoh 2008; Hoff et al. 2016; Iwata and Gotoh 2012; Lomsadze et al. 2005; Stanke et al. 2006, 2008). Arthropoda protein database from OrthoDB v12 was provided (Kuznetsov et al. 2023). Then, we used InterProScan (v.5.76‐107.0) (Zdobnov and Apweiler 2001) to do functional annotation and assign GO terms to all predicted protein‐coding genes. Candidate genes were identified by mapping the high‐confidence concordant SNPs to their respective gene models. Enrichment tests were conducted separately for SNPs associated with each environmental predictor. We used the topGO (v2.60.1) R package to test for enrichment within the Biological Process (BP) ontology. To account for the hierarchical structure of GO terms and minimize redundancy, we applied the ‘weight01’ algorithm combined with Fisher's exact test. *p*‐values were adjusted for multiple testing using a significance threshold of FDR < 0.05. Finally, we extracted the specific candidate genes associated with each enriched GO term to identify functional clusters related to climate adaptation.

We fit a constrained RDA model on the full genotype matrix of high‐confidence concordant candidates to quantify genetic offset. Genetic offset was measured between current and future climates for each landscape pixel as the Euclidean distance in RDA space between the genotypic composition predicted under current climate and that predicted under future climate (Gain et al. 2023). Landscape‐level offsets were measured with two emission pathways (SSP2‐4.5 and SSP5‐8.5) and three time periods (2041–2060, 2061–2080, 2081–2100) using future climatic data from WorldClim 2 (BCC‐CSM2‐MR general circulation model) (Fick and Hijmans 2017). Population offsets were summarized as the mean of individual offsets at each sampling locality. To identify potential genetic rescue donor sites, we adapted the landscape genomics prediction framework (Fitzpatrick and Keller 2015) using a jackknife‐based ordination approach (*n* = 20). Briefly, for each jackknife replicate, a distance‐based RDA was fit on a correlation‐based genetic distance matrix, according to population structure PCs (*K* = 4). Site scores on the first four constrained axes (genomic principal components, gPCs) were then regressed against climate predictors via a second RDA and used to predict genomic composition across all landscape pixels under current climate. Mean predicted gPCs were calculated across stable jackknife replicates (defined as the 90% of pixels with lowest prediction variance across replicates). A 90th‐percentile pairwise Euclidean distance among landscape gPC predictions served as a normalizing scale factor. For each population, the future genomic (SSP5‐8.5 2061–2080) requirements were extracted at the population centroid. The genomic mismatch between this target and every current landscape pixel was then computed as the scaled Euclidean distance in gPC space (Rellstab et al. 2021), and inverted to yield a donor suitability surface. Pixels with high suitability values were considered to contain genetic compositions already adapted to projected future climatic conditions and were therefore identified as priority sources for assisted gene flow. Because this distance is normalized by the 90th‐percentile pairwise distance among landscape gPC predictions, mismatch is a dimensionless quantity bounded near zero for an ideal donor (genomic composition identical to the recipient's projected future requirement) and increasing as genomic dissimilarity grows relative to the total genomic variation across the study region. Mismatch values are therefore interpreted on a relative scale, with lower values indicating more suitable donor sources.

## 3 Results

### 3.1 Population Structure and Gene Flow

PCA of SNPs revealed a highly structured genetic landscape, with the first three components (PC1, PC2 and PC3) explaining 9.9%, 6.5% and 5.7% of the total variance, respectively (Figure S2). While no single pairwise combination of PCs accounted for a majority of the variance, PC1–PC3 collectively resolved the major geographic lineages. PC1 primarily separated the Utah samples from all other populations. PC2 further differentiated the Mogollon Rim populations from the southern sky island complexes. Within the PC1–PC3 space, the two primary sky island localities, Mt. Lemmon and Chiricahua Mt., were clearly separated. Single representatives from Cochise County, Mt. Graham and Mt. Wrighton occupied intermediate positions in the PCA space. Samples from Mexico and individuals of unknown origin formed a distinct cluster, separated from both the Mogollon Rim and sky island populations. A three‐dimensional projection of the first three PCs further highlighted the hierarchical separation among Utah, the Mogollon Rim, the sky island localities and the Mexico/unknown group (Figure 2A).

The ADMIXTURE identified *K* = 2 as the model with the lowest CV error (Table S2). However, this level of clustering provided limited biological resolution compared to the structure inferred from the PCA. We therefore examined higher *K* values, focusing on *K* = 4, which produced ancestry components that better aligned with geographic distributions by separating Utah, the Mogollon Rim, the sky islands and the Mexico/Unknown groups (Figure 2B). At *K* = 4, Chiricahua Mt. and Mt. Lemmon were dominated by distinct ancestry components, while individuals represented by single samples from the broader sky island region (e.g., Mt. Graham, Mt. Wrighton) displayed mixed ancestry proportions. Mexico and the unknown‐origin individuals showed nearly identical ancestry profiles (Figure 2B).

Our *D*‐statistic analyses revealed only weak signals of allele sharing among populations, with uniformly small *D*‐values (*D* < 0.02) across all trio combinations (Table S4). The Mt. Lemmon population exhibited significant gene flow signals with multiple populations, including Chiricahua Mt. (*D* = 0.0138, *Z* = 3.73, *p* = 0.0002), Mt. Graham (*D* = 0.0176, *Z* = 3.95, *p* = 0.0001), Mogollon Rim (*D* = 0.0072, *Z* = 3.71, *p* = 0.0002) and Mt. Wrighton (*D* = 0.0129, *Z* = 2.75, *p* = 0.0059). Additionally, we detected significant gene flow between Chiricahua Mt. and Mogollon Rim (*D* = 0.0077, *Z* = 3.50, *p* = 0.0005), as well as between Mt. Graham and Mogollon Rim when tested through different population combinations (*D* = 0.0065–0.0066, *Z* = 2.42–2.54, *p* = 0.0112–0.0155). Because the largest signals depended on the single historical Cochise sample and other single‐individual localities, we repeated the analysis using recent samples only (Table S5). In this analysis, we detected significant excess allele sharing between Chiricahua Mt. and Mogollon Rim when compared to Mt. Graham (*D* = 0.0065, *Z* = 2.54, *p* = 0.0112; *f*_4_‐ratio = 0.0992). Similarly, Mt. Lemmon showed excess sharing with Mogollon Rim when compared to Mt. Graham (*D* = 0.0066, *Z* = 2.42, *p* = 0.0155; *f*_4_‐ratio = 0.0997). When Mt. Lemmon and Chiricahua Mt. were directly compared for Mogollon ancestry, they showed no significant differences (*D* = 0.0004, *Z* = 0.2982, *p* = 0.7655). All other population pairs showed no significant gene flow (*p* > 0.05; Table S5). The significant signals consistently involved single‐individual localities (Mt. Graham, Mt. Wrighton, Cochise), for which limited sampling precludes distinguishing contemporary gene flow from ancestral structure or ghost introgression. Among the four well‐sampled lineages, *D*‐statistics were small and non‐significant, and *f*_4_‐ratios were correspondingly low, indicating little to no detectable gene flow among the lineages used in demographic modelling. On this basis, we fixed migration to zero in our subsequent δaδi models. This interpretation is further supported by the significant positive correlation between genetic and geographic distance (Mantel's *r* = 0.79, *R* ^2^ = 0.62, *p* < 0.0001; Figure 2C), indicating that genetic differentiation is structured primarily by geographic distance among mountain ranges. This pattern suggests that gene flow is geographically restricted among mountain ranges.

While the PCA and ADMIXTURE included all available specimens to provide a broad geographic survey, including singletons and samples of unknown origin reduced the resolution of population‐specific parameters. Consequently, we focused our subsequent investigation of divergence and demographic history on the four well‐defined lineages (Mogollon Rim, Mt. Lemmon, Chiricahua Mt. and Utah) identified above.

### 3.2 Demographic History and Coalescent Modelling

**FIGURE 3:**
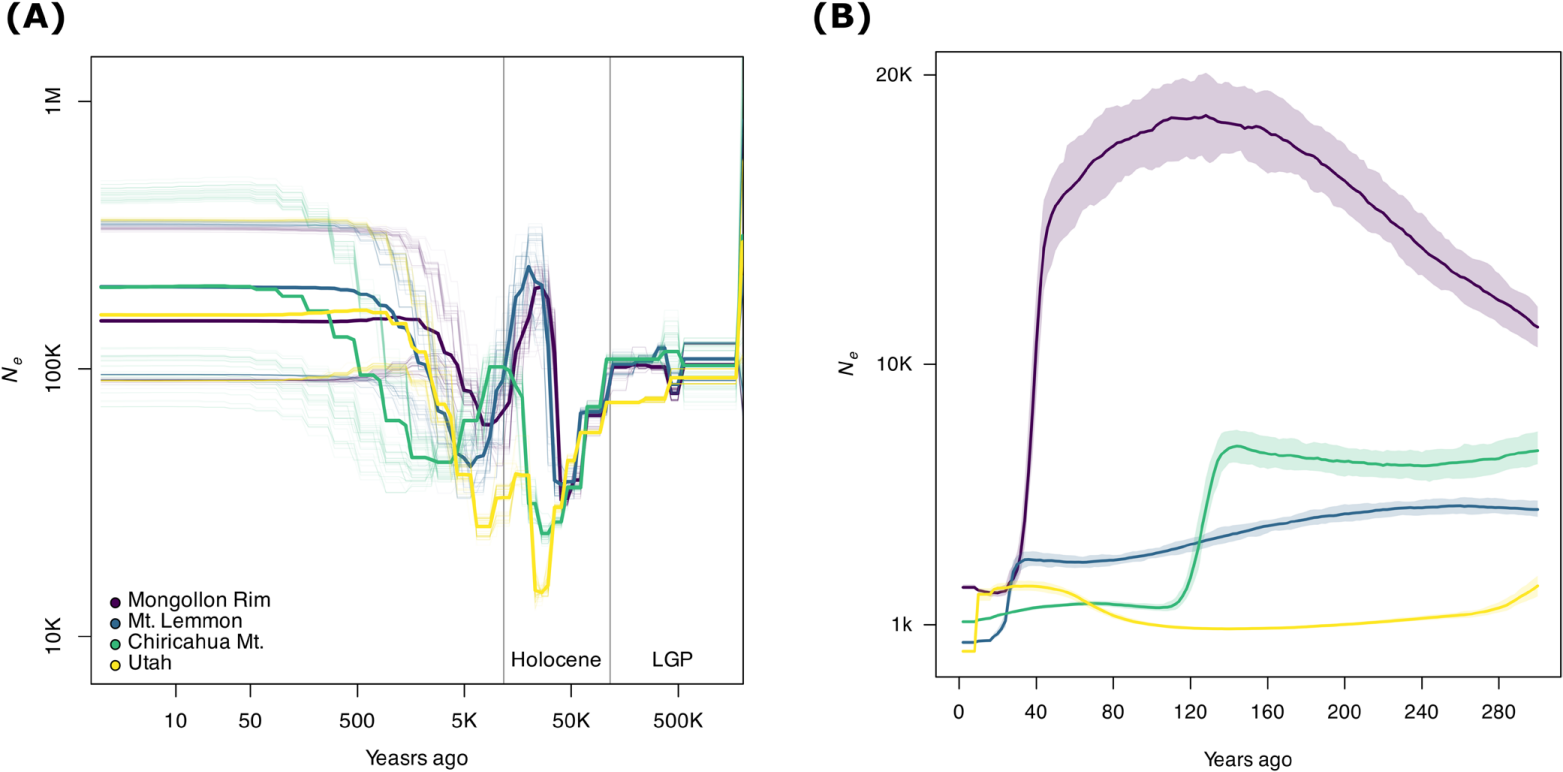
Demographic history of *D. grantii*. (A) Inferred by SMC++. Log‐scale plot showing *N*_e_ trajectories through time for four *D. grantii* lineages. Mogollon Rim, Mt. Lemmon, Chiricahua Mt. and Utah. Light shaded bands around each trajectory represent 95% confidence intervals from 100 independent SMC++ iterations per population. (B) Inferred by GONE2. Fine‐scale *N*_e_ estimates for the four *D. grantii* lineages over the most recent 300 years (150 generations, assuming 2‐year generation time). Solid coloured lines represent point estimates, and shaded regions indicate 95% confidence intervals.

The sensitivity analysis confirmed that varying the mutation rate rescaled both the magnitude of *N*_e_ and the timing of demographic events, while varying generation time rescaled only the timing (Figure S3). We therefore interpret the relative differences among lineages and the overall demographic pattern as robust, while treating the absolute *N*_e_ values and event ages as approximate. The SMC++ analysis revealed highly synchronous demographic trajectories across all four primary lineages, characterized by dramatic fluctuations in *N*_e_ throughout the late Pleistocene and Holocene (Figure 3A). Historically, all populations experienced a significant expansion during the Last Glacial Period (LGP), peaking approximately 10,000–20,000 years ago. Following this peak, all lineages underwent a rapid and severe population contraction coinciding with the onset of the Holocene. The Utah population experienced the most severe bottleneck, reaching the lowest *N*_e_ among all sampled groups during this period. In the more recent past (within the last 300 years), all populations have shown signs of recovery and stabilization, though they maintain distinct *N*_e_ levels (Figure 3B). The Mogollon Rim population shows a pronounced increase in *N*_e_ beginning approximately 30–40 generations ago, reaching a peak followed by a gradual decline toward the present, though *N*_e_ remains substantially higher than in other populations. In contrast, Mt. Lemmon exhibits a more modest but steady increase in *N*_e_ over time, suggesting gradual recovery rather than a sharp demographic shift. The Chiricahua Mt. population displays an expansion beginning around 120–130 generations ago, stabilizing at intermediate *N*_e_ values thereafter. Finally, the Utah population maintains consistently low *N*_e_ across the time series, with only a slight recent increase, indicating limited evidence for substantial demographic recovery.

Demographic modelling supported isolation with asymmetric population size change (ISC model) as the best‐fit model across all six pairwise comparisons (Table S3). However, AIC differences between ISC and SI were small (ΔAIC = 0.02–0.45). Despite this, parameter estimates were highly consistent across independent comparisons. This suggests that the primary signal in the data is one of simple isolation rather than size change. The improvement of ISC over SI therefore reflects the added flexibility of the size‐change parameters rather than a fundamentally different demographic scenario, and both models converge on the same conclusion, which is that these lineages diverged in allopatry without detectable post‐divergence gene flow. Utah showed contraction to 18%–28% of ancestral size in all comparisons, while Mogollon Rim and Mt. Lemmon showed expansion to 3–51× ancestral size (Table S3). Divergence times were shallow across all pairs (*T* = 0.024–0.134 in units of 2 × *N*_ref_ generations). Chiricahua Mt./Utah and Mt. Lemmon/Chiricahua Mt. pairs have the deepest and shallowest divergence, respectively (*T* = 0.134; *T* = 0.024).

### 3.3 Patterns of Genomic Divergence

**FIGURE 4:**
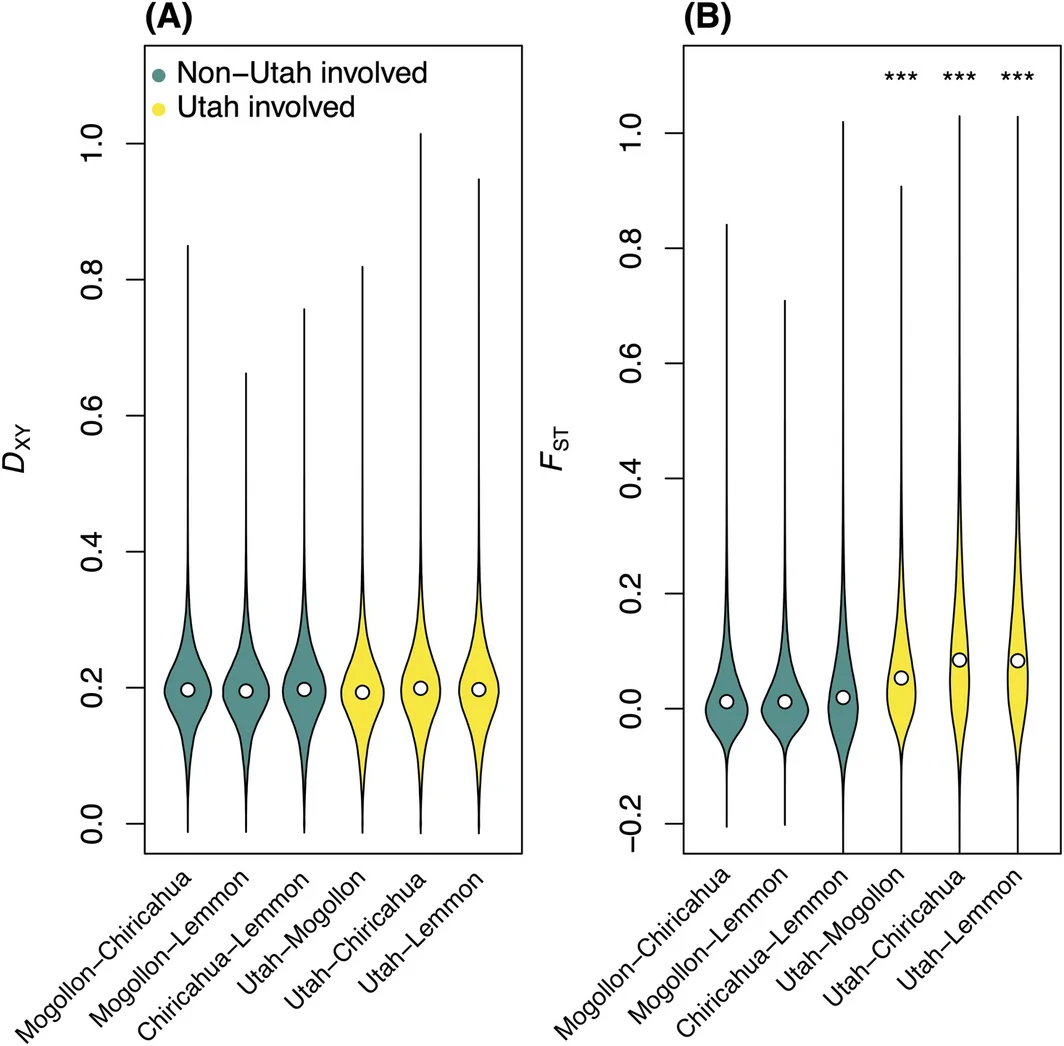
*F*_ST_ vs. *D*_*XY*_ distributions distinguish drift from divergence. (A) *D*_*XY*_ shows no significant difference between Utah‐involved and non‐Utah population pairs (Mann–Whitney *U*, *p* = 0.33), indicating congruent divergence times. (B) *F*_ST_ distributions reveal significantly higher differentiation in Utah comparisons (Mann–Whitney *U*, *p* < 2.2 × 10^−16^), supporting a model where high *F*_ST_ reflects drift‐driven allele fixation rather than deep divergence. Together, these results indicate Utah's genetic distinctiveness results from demographic history, not uniquely ancient isolation.

To test the hypothesis that the Utah population has undergone accelerated genetic drift, we compared genomic window distributions of *F*_ST_ and *D*_*XY*_ between the Utah population and each of the other sampled populations. A one‐sided Mann–Whitney *U* test confirmed that *F*_ST_ is significantly higher in comparisons involving Utah (*W* = 3.8575 × 10^10^, *p* < 2.2 × 10^−16^), consistent with increased levels of allelic fixation. In contrast, the *D*_*XY*_ distributions revealed no significant difference between the two groups (*W* = 6.2203 × 10^10^, *p* = 0.3342), supporting a model of congruent divergence times across all lineages despite varying levels of contemporary genetic drift (Figure 4).

**FIGURE 5:**
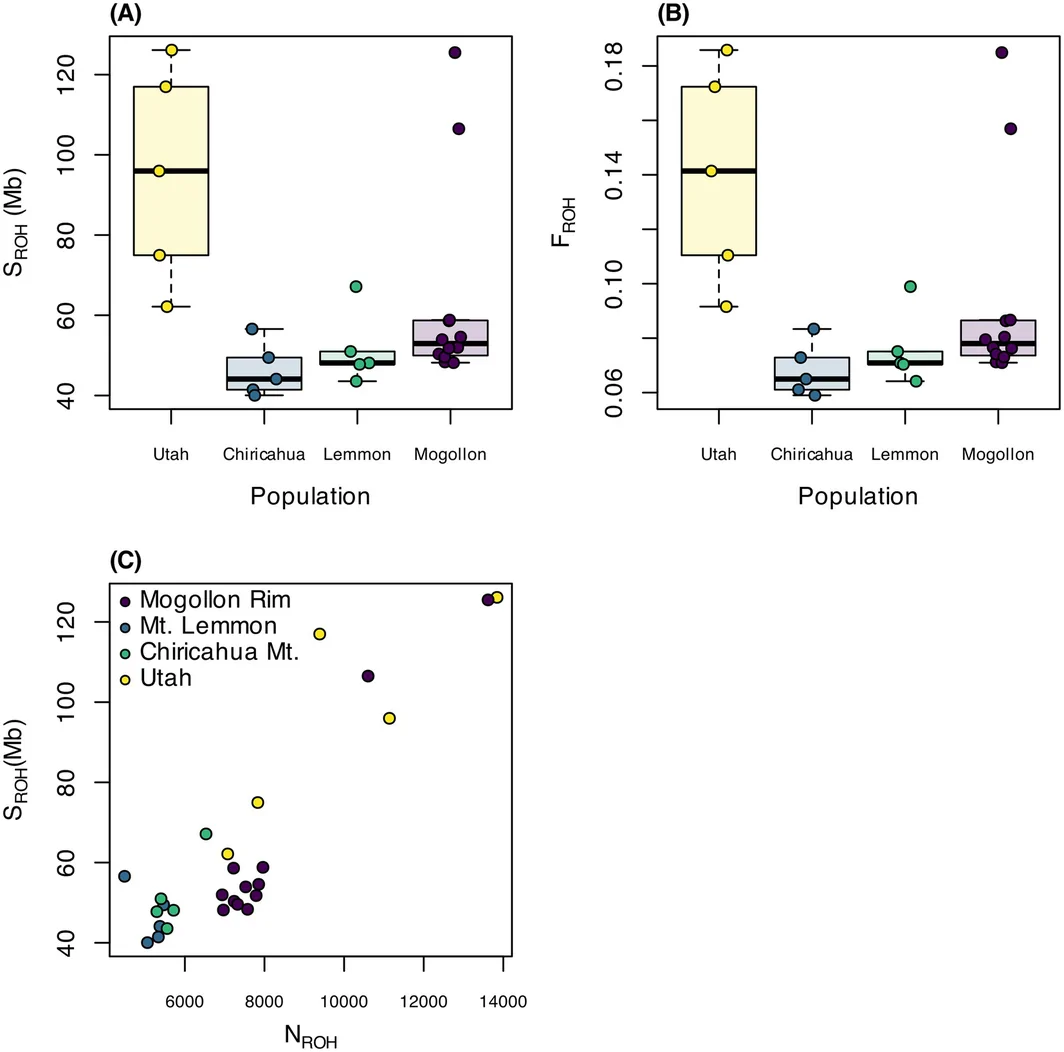
Runs of homozygosity and inbreeding coefficients reveal genetic load accumulation in isolated populations. (A, B) Distribution of the sum of ROH (S_ROH_, sum in Mb) and inbreeding coefficient calculated by ROH (F_ROH_) by population. Utah exhibits elevated S_ROH_ and F_ROH_ than other lineages. (C) Total number of ROH (N_ROH_) correlates with S_ROH_ show that smaller, isolated populations accumulate more frequent ROH, consistent with elevated inbreeding and genetic drift.

ROH segments in all populations were predominantly short (< 0.1 Mb), with no long ROH (> 5 Mb) detected in any individual (Figure S4). ROH analysis shows significant differences in S_ROH_ across populations (Kruskal–Wallis: S_ROH_ *χ* ^2^ = 13.26, *p* = 0.004; F_ROH_ *χ* ^2^ = 13.26, *p* = 0.004; N_ROH_ *χ* ^2^ = 19.43, *p* = 0.0002). The Utah population exhibited substantially elevated total ROH length and inbreeding coefficients (median S_ROH_ ~ 96.0 Mb; median F_ROH_ ~ 0.140) compared to Chiricahua Mt. (S_ROH_ ~ 44.1 Mb, F_ROH_ ~ 0.065), Mt. Lemmon (S_ROH_ ~ 48.1 Mb, F_ROH_ ~ 0.076) and Mogollon Rim (S_ROH_ ~ 52.9 Mb, F_ROH_ ~ 0.071) (Figure 5). Pairwise comparisons identified significant differences between Utah and Chiricahua Mt. (Wilcoxon, *p* = 0.048).

### 3.4 Genomic Environmental Association (GEA)

**FIGURE 6:**
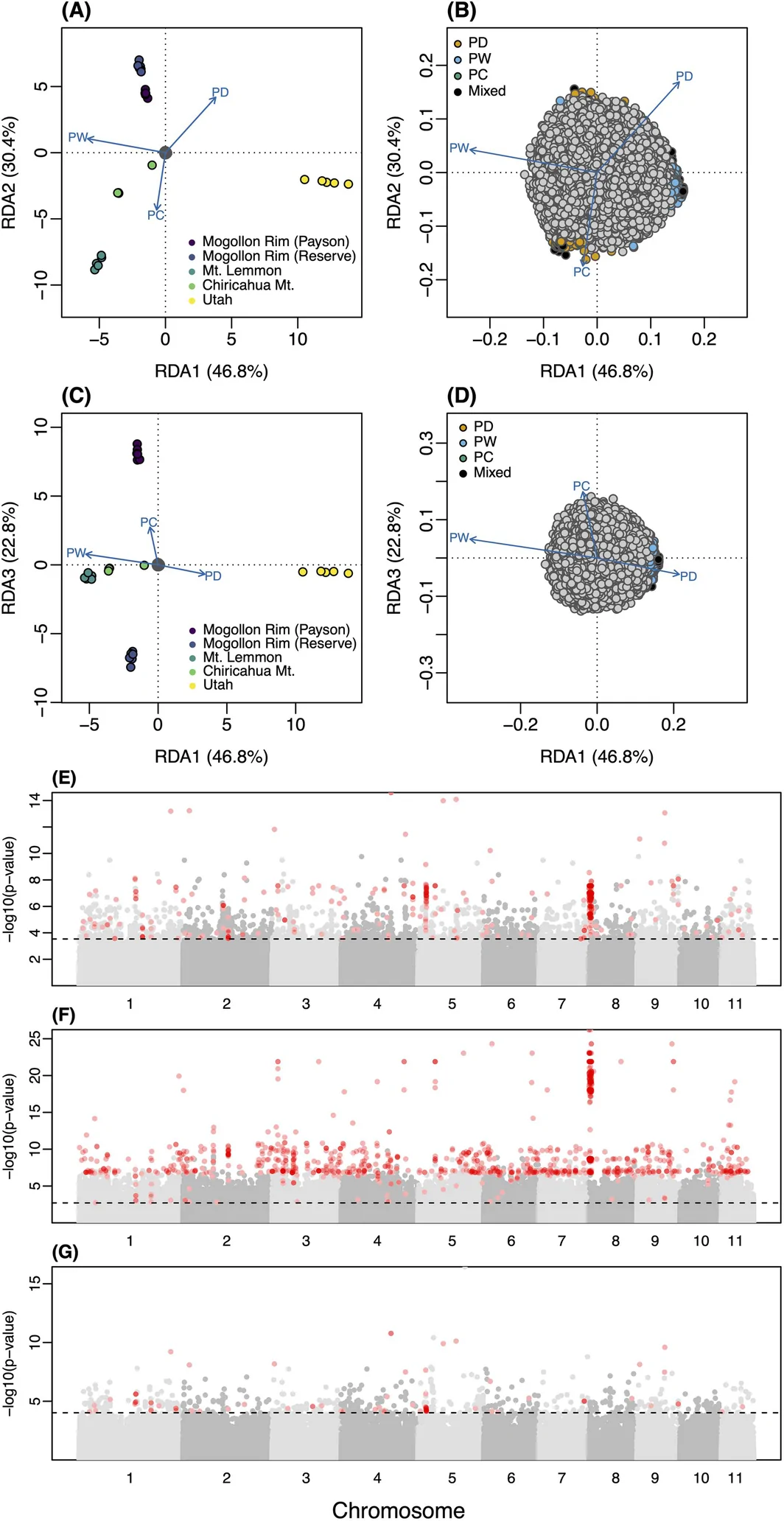
Redundancy Analysis (RDA) of population genetic structure and biometric variation. (A, C) RDA biplots show the first three constrained axes (RDA1, RDA2 and RDA3) with population samples coloured by geographic origin. Arrows indicate biometric variables that constrain the ordination, with arrow direction and magnitude showing the strength of association with each RDA axis. (B, D) Zoomed views of the SNP space showing individual genotypes in relation to the RDA axes with significantly associated SNPs highlighted and coloured by their associated precipitation variables (Precipitation of the Driest Quarter (PD), Precipitation of the Wettest Quarter (PW), Precipitation of the Coldest Quarter (PC) or Mixed (SNPs associated with more than one variable)). Manhattan plots showing LFMM‐significant SNPs and RDA‐LFMM concordance across the genome. (E) PD, (F) PW and (G) PC. The *y*‐axis shows –log_10_ (*p*‐value) with the dashed horizontal line indicating the FDR significance threshold (FDR < 0.05). Of the 20,867 candidate SNPs identified across all three variables, PW was associated with 17,448 loci, followed by PD (2571) and PC (848). Coloured points mark SNPs that are significantly associated by LFMM (above the threshold) and represent the intersection of LFMM and RDA analyses. The overlap between RDA and LFMM methods identified 1367 high‐confidence concordant SNPs.

The RDA model was significant (Permutation test, *p* < 0.001) and explained a substantial portion of the genomic variation across the four primary lineages (Adjusted *R* ^2^ = 0.063). Of the total constrained variance, the first two axes accounted for 77.16% of the explained variation (RDA1 = 46.77%; RDA2 = 30.39%) (Figure 6). The high eigenvalues for the first two axes suggest that the climate variables used, PD, PW and PC, capture distinct environmental gradients that vary across the sky islands and the Mogollon Rim. Using a ±3 SD threshold on these two axes, we identified 1411 outlier SNPs. The LFMM2 analysis, which accounts for the four‐way population structure (*K* = 4), identified a significant number of SNPs associated with regional precipitation gradients. We found a total of 20,867 unique candidate SNPs across the three variables at an FDR threshold of 0.05 (Figure 6). PW was associated with the vast majority of these loci (*n* = 17,448), followed by PD and PC, which were associated with 2571 and 848 SNPs, respectively. The intersection of these two methods yielded a highly robust set of 1367 high‐confidence concordant SNPs (Figure 6). This represents a 96.8% overlap of the RDA outliers with the LFMM results. GO enrichment analysis of the high‐confidence concordant SNPs revealed significant biological themes associated with precipitation seasonality. For PD and PW, we identified a total of seven significantly enriched biological process terms (FDR < 0.05) (Table 1). In contrast, no significant enrichment was detected for loci associated with PC.

**TABLE 1:** GO enrichment analysis of concordant SNPs associated with precipitation variables. Significantly enriched biological process terms (FDR < 0.05) identified for loci associated with precipitation seasonality. Seven enriched terms were identified for PW and PD, while no significant enrichment was detected for PC.

GO.ID	Annotated	Significant	Expected	Fisher	FDR
*Precipitation of Wettest Quarter*					
GO:0030866^a^	12	2	0.07	0.002	0.04071429
GO:0006491^b^	13	2	0.07	0.0024	0.04071429
GO:1902004^c^	1	1	0.01	0.0057	0.04071429
GO:0045217^d^	1	1	0.01	0.0057	0.04071429
GO:0009953^e^	1	1	0.01	0.0057	0.04071429
GO:0070373^f^	1	1	0.01	0.0057	0.04071429
GO:0046426^g^	1	1	0.01	0.0057	0.04071429
*Precipitation of Driest Quarter*					
GO:0006491^b^	13	2	0.02	0.00027	0.0135

^a^ Cortical actin cytoskeleton organization.

^b^ N‐glycan processing.

^c^ Positive regulation of amyloid‐beta formation.

^d^ Cell–cell junction maintenance.

^e^ Dorsal/ventral pattern formation.

^f^ Negative regulation of ERK1 and ERK2 cascade.

^g^ Negative regulation of receptor signalling pathway via JAK–STAT.

**FIGURE 7:**
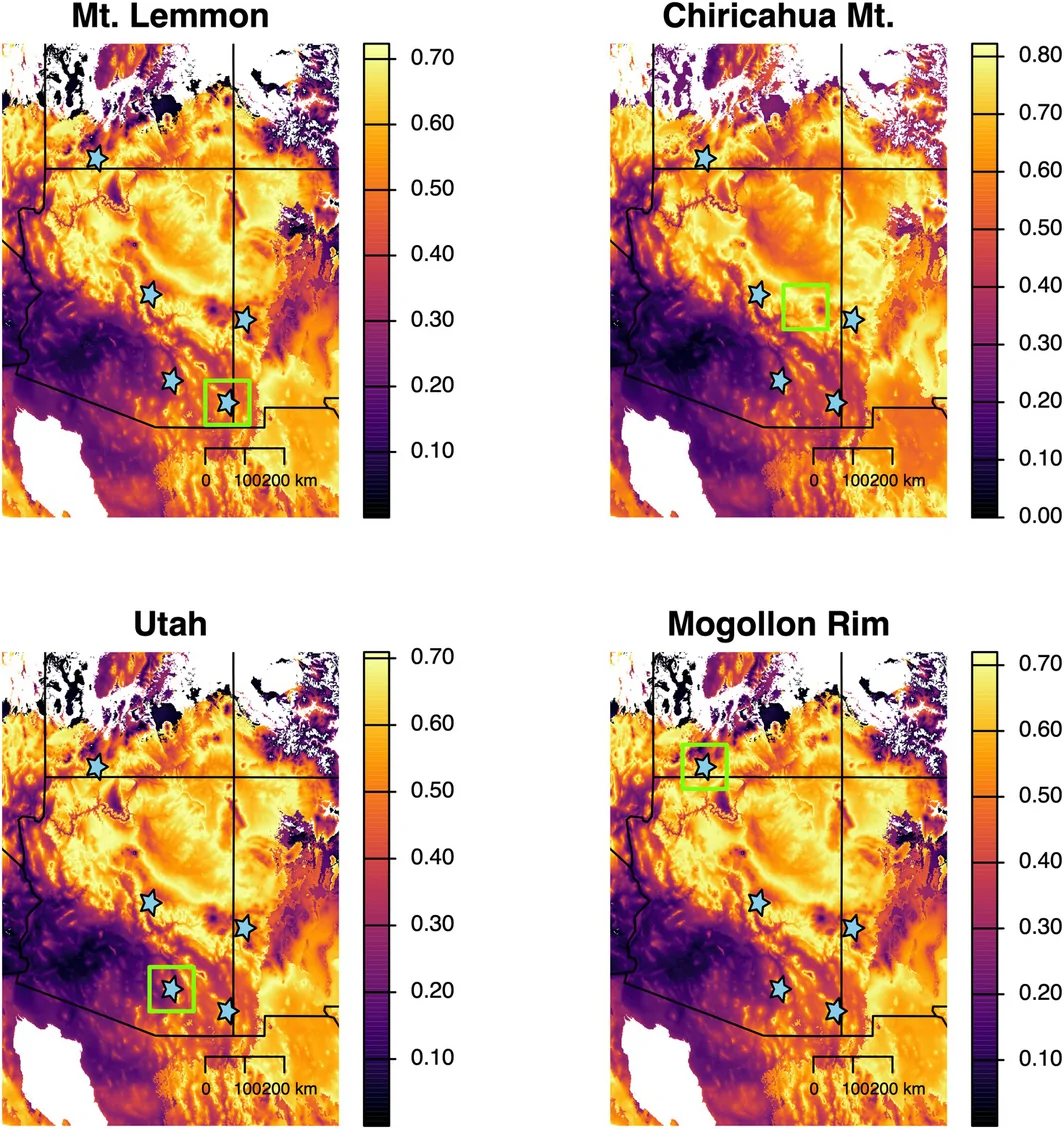
Genetic rescue donor identification maps for assisted gene flow. Maps show genomic compatibility between each population and potential donor regions under future climate conditions (SSP5‐8.52061–2080). Higher numbers indicate better genetic matches for facilitating adaptive gene flow. Stars mark population locations, and boxed regions highlight the identified optimal donor sources.

Genetic offset varied among populations across all six climate scenarios we tested. Mt. Lemmon was the most vulnerable population, and it had the highest offset values in every scenario (SSP2‐4.5/SSP5‐8.52041/2061/2081) (9291,055 units) (Table S6). The other three populations showed more moderate and broadly similar offset values (589–682 units). Genetic rescue donor site analysis identified potential source populations for assisted gene flow for each population under the SSP5‐8.5 2061–2080 scenario. Mt. Lemmon showed its best genomic donor match near the Chiricahua Mt. region (mismatch = 0.0006, on a scale where 0 indicates a genomically ideal donor and values increase with genomic dissimilarity). The best donor source for Chiricahua Mt. corresponded to the Mogollon Rim region (mismatch = 0.0001), while Mogollon Rim populations would be best supplemented with individuals sourced from the Utah region (mismatch = 0.0003). Utah's best donor match fell within the southern sky island region near Mt. Lemmon (mismatch = 0.0003) (Figure 7).

## 4 Discussion

In this study, we used whole genome sequencing across different populations of *D. grantii* to reconstruct its evolutionary history and evaluate the genetics of populations across the sky island archipelago. Our analyses show that (1) genetic variation is strongly geographically structured, with differentiation shaped primarily by isolation‐by‐distance and only geographically restricted historical gene flow, (2) all four lineages share a synchronous history of Last Glacial Period expansion followed by severe Holocene contraction, with the peripheral Utah lineage showing the lowest *N*_e_ and the Mogollon Rim retaining the largest, (3) precipitation seasonality is associated with candidate loci underlying local adaptation, and (4) projected climate change is predicted to affect populations unevenly (Mt. Lemmon as the most vulnerable population among all). These results reconstruct the demographic and adaptive history of a sky island insect while identifying the Mogollon Rim as a potential reservoir of standing genetic variation for conservation.

### 4.1 Resolving Evolutionary Lineages

Our results show that the strong genomic structure in *D. grantii* can be used to help assign specimens with missing or ambiguous locality metadata. Even when precise collection information was unavailable for some museum and private‐collection individuals, their placement in PCA space and their ADMIXTURE ancestry profiles provided a consistent basis for inferring likely geographic origin. For example, the Mexico and Unknown individuals formed a distinct genetic cluster that was clearly separated from both the Mogollon Rim and the sky island populations, suggesting that the unknown‐locality specimens were collected from the same region as the Mexico sample (Figure 2). The Cochise County specimen presents a more ambiguous case. Cochise County includes the Chiricahua Mt. but not Mt. Lemmon, and in the 3D PCA it appears intermediate rather than clustering cleanly with either Chiricahua Mt. or Mt. Lemmon (Figure 2A). This placement could reflect incomplete lineage sorting, admixture or simply limited sampling of nearby sky island ranges. Similarly, the single individuals from Mt. Graham and Mt. Wrightson, which is geographically positioned between Mt. Lemmon, the Chiricahua Mt. and the Mogollon Rim, also fall in an intermediate position in the 3D PCA (Figure 2A) and show mixed ancestry components in ADMIXTURE (Figure 2A). In addition to contemporary samples, our genomic data showed the applicability of the approach to help with the assignment of unidentified or museum specimens with uncertain or missing locality data, which supports the broader utility of this framework that integrates evolutionary history, biodiversity conservation and natural history collections using the same genomic dataset.

Significant *D*‐statistic and *f*_4_‐ratio estimates indicate that Mogollon Rim has received approximately equal amounts of ancestry from both Mt. Lemmon and Chiricahua Mt. (Tables S4 and S5), consistent with ADMIXTURE results (Figure 2A, *K* = 4). Mogollon Rim is an admixed population containing ancestry components from both populations while Chiricahua Mt. and Mt. Lemmon populations remain largely pure. We also detect excess allele sharing between Mt. Lemmon and Mt. Graham; but with only one Mt. Graham individual, our power to detect gene flow was limited to scenarios with > 15%–20% admixture. In contrast, ADMIXTURE uses model‐based clustering across all SNPs and can detect weaker signals, particularly when admixture is ancient and distributed across many small ancestry blocks. The ADMIXTURE signal in Mt. Graham may therefore reflect: (1) ancient gene flow that has been diluted through recombination over many generations, making it difficult to detect with ABBA‐BABA; (2) low‐level contemporary gene flow (< 5%–10%) from multiple sources that collectively creates a complex ancestry profile but individually falls below ABBA‐BABA detection thresholds; or (3) phylogenetic structure where Mt. Graham shares alleles with multiple populations due to ancestral relationships rather than recent gene flow.

Mt. Graham and other sparsely sampled localities may represent junctions in regional connectivity or zones where admixed genotypes persist. However, because these localities are represented by only one individual each, we cannot formally test with strong statistical power whether they represent active hybridization zones, ongoing gene flow or simply within‐population variation. Biologically, the pattern of admixture detected in Mogollon Rim is consistent with historical connectivity among southern sky island ranges during favourable climatic windows, potentially via stepping‐stone dispersal across intervening low‐elevation valleys (Warshall 1995). This interpretation is supported by the significant isolation‐by‐distance signal observed within the southern population complex (Mantel's *r* = 0.79, *p* < 0.0001), indicating that gene flow has been geographically structured by distance even when connectivity was present. An alternative explanation for these complex admixture patterns is introgression from unsampled or extinct ‘ghost’ populations (Lawson et al. 2018). Ghost introgression occurs when genetic signals are misattributed to sampled populations but actually originate from unsampled sources, including geographically unsampled, temporally extinct or represent cryptic lineages. Under this scenario, Chiricahua Mt./Mt. Lemmon admixture signal in Mogollon Rim could alternatively represent introgression from a ghost population that was itself intermediate between eastern and western lineages, while Mt. Graham's multi‐source ADMIXTURE profile might reflect ancestry from an ancient, unsampled sky island population rather than contemporary gene flow from multiple directions. Distinguishing between contemporary stepping‐stone gene flow and ghost introgression requires denser geographic sampling across the sky island archipelago, particularly in intervening ranges that could harbour cryptic or unsampled populations.

The observed discordance between high *F*_ST_ and stable *D*_*XY*_ in the Utah population suggests that its genetic phenomenon is a product of accelerated drift rather than evolutionary independence divergence. While the Utah population exhibited the highest *F*_ST_ in all pairwise comparisons, the absolute divergence *D*_*XY*_ was not significantly different from that of the more connected southern populations. Because *F*_ST_ is a relative measure that is strongly influenced by within‐population diversity and therefore sensitive to reductions in *N*_e_, it can increase rapidly in populations that have experienced bottlenecks or persist at small *N*_e_. In contrast, *D*_*XY*_ reflects the absolute number of differences between populations and is generally expected to track deeper‐time separation more directly, making it less responsive to short‐term changes in *N*_e_ than relative metrics. Under these circumstances, high *F*_ST_ with unchanged *D*_*XY*_ is expected when divergence is driven primarily by loss of variation and allele fixation within a small isolate rather than by the accumulation of substitutions over a long period of independent evolution.

### 4.2 Demography and Source‐Sink Connectivity

Because SMC++ and GONE2 rely on different genomic signals and resolve different timescales, their *N*_e_ estimates are not expected to match in the most recent window. SMC++ draws on the site frequency spectrum and linkage information across deep time, and GONE2 uses LD to infer recent *N*_e_ over the last ~300 years. The confidence intervals shown for each method are not absolute confidence in the recent estimates or cross‐method agreement. The SMC++ intervals reflect variance across SMC++ iterations which capture uncertainty in the estimation procedure. Each method has its own assumptions, which explains how the two can diverge in the recent window while each shows relatively tight intervals. Despite this, the two approaches are consistent on the extremes of the *N*_e_ ranking, with the Mogollon Rim lineage consistently showing the largest *N*_e_, whereas Utah consistently shows the lowest. We interpret this shared pattern as a robust feature of the data, while treating the relative ordering of the intermediate lineages as method‐dependent.

The synchronous post‐glacial declines inferred across lineages underscore the sensitivity of *D. grantii* to late‐Quaternary climate shifts, a pattern widely reported in montane and cool‐adapted taxa (Garg et al. 2020; Wiens et al. 2019), where climatic warming drives upslope range shifts, elevational habitat loss and fragmentation into sky island mountain ranges. Our results are consistent with *D. grantii* having been more geographically continuous during glacial climates, with modern populations representing fragmented remnants of a formerly broader distribution. As shown in our sensitivity analysis (Figure S3), plausible variation in these parameters shifts the absolute magnitude and timing of *N*_e_ estimates, although the qualitative pattern (glacial expansion followed by Holocene contraction) is robust. The Utah lineage underwent the most severe contraction and maintained the lowest inferred *N*_e_ throughout the Holocene, supporting the expectation that peripheral populations are especially vulnerable to demographic collapse under climate‐driven habitat loss. In contrast, the Mogollon Rim lineage consistently maintained larger *N*_e_ than the isolated sky island and Utah populations. This pattern matches the biogeography of the region. The Mogollon Rim provides a relatively continuous belt of montane forest habitat, whereas sky island ranges are separated by arid lowlands that restrict dispersal and promote drift in small populations. From an applied perspective, these results suggest that the Mogollon Rim may function as a central reservoir of standing genetic variation, while smaller, high‐drift populations may be more vulnerable to environmental stochasticity and future warming. Management efforts that maintain the integrity and connectivity of Mogollon Rim forests (and reduce further fragmentation) could therefore yield disproportionate benefits for preserving regional evolutionary potential, particularly if future climate trajectories further isolate already‐small peripheral populations (Fleischner et al. 2024).

Our results demonstrate the value of combining a reference genome with whole‐genome resequencing using populations sampled across space and time for biodiversity research and conservation applications. Specifically, while a previous genome‐wide SNP study similarly detected a post‐Pleistocene decline in *N*_e_ in the Mogollon Rim population (Huang 2019), our expanded dataset using the same sampled individuals not only recovered this same pattern but also revealed additional historical demographic fluctuations, as indicated by the SMC++ analyses, as well as a more recent and severe population decline. These finer‐scale demographic changes were likely not detectable previously because of limited statistical power. We further found differences in F_ROH_ and S_ROH_ (Figure 5) between isolated population and larger population, providing additional support for ongoing demographic decline and elevated inbreeding in small, isolated sky island populations. The higher F_ROH_ values in isolated populations indicate increased recent inbreeding, consistent with the severe contemporary bottlenecks detected by SMC++, while lower F_ROH_ in larger populations like Mogollon Rim suggests they have maintained larger *N*_e_ and lower inbreeding despite historical fluctuations. These patterns underscore the conservation vulnerability of isolated sky island populations and highlight the utility of whole‐genome data for detecting subtle but critical demographic signals that inform management priorities.

### 4.3 The Genomic Architecture of Climate Adaptation

Our findings reveal that precipitation seasonality, particularly the wettest of the summer monsoon quarter, represents the strongest axis of adaptive evolution in *D. grantii*. Precipitation of the wettest quarter (PW) is associated with over 17,000 significant SNPs. This signal is robust across independent analytical approaches, with nearly 97% of RDA‐identified outliers confirmed by LFMM analysis. This reduces the likelihood that demographic artefacts are the primary driver of these patterns. This convergence is consistent with polygenic signatures of local adaptation to monsoon‐driven moisture regimes. The Go enrichment terms further support this interpretation by identifying cellular mechanisms directly relevant to moisture stress: genes involved in cytoskeletal organization, glycoprotein processing, cell–cell junctions and signalling regulation (Table 1). These pathways suggest that selection has shaped physiological traits, such as barrier integrity and desiccation resistance. These are essential for surviving the predictable but intense seasonal swings in rainfall in these montane environments. Our results thus indicate that monsoon seasonality is the primary adaptive landscape feature shaping genomic divergence in this species, with winter precipitation playing a comparatively minor role in driving evolutionary change.

Translating these adaptive signatures into projections of future vulnerability, our genetic offset analyses identified Mt. Lemmon as the most at‐risk population, whereas the Mogollon Rim is best positioned in the long term. Its relatively continuous montane forest habitat has allowed it to maintain the largest *N*_e_ and thus the greatest reservoir of standing genetic variation to fuel future adaptation. Donor‐site analyses further pointed to specific candidate sources for assisted gene flow. Notably, all four best‐match mismatch values were very small and closely clustered (0.0001–0.0006), suggesting that the lineages retain broadly compatible genomic compositions and that no single population is a uniquely poor donor. This pattern of low mismatch advises that these populations could serve as mutual sources of adaptive variation, though the small magnitude of these differences means such rankings should be treated cautiously.

However, genetic offset predictions have assumptions that need careful interpretation. It assumes that candidate loci capture the genomic basis of local adaptation, remain stable through time, and no ongoing gene flow. These conditions are difficult to meet in practice, and that offset values are best understood as relative rather than absolute measures of risk (Láruson et al. 2022; Rellstab et al. 2021). Yet, a recent common‐garden experiment in a marine oyster showed that genetic offset may poorly predict future survival (Rumberger et al. 2026). Under those circumstances, we treat our vulnerability rankings and donor‐site identifications as testable hypotheses for prioritization rather than definitive predictions. A successful genetic assistant program requires independent lines of evidence, such as common‐garden or reciprocal‐transplant experiments, fitness assays across environmental gradients, and denser geographic and temporal sampling to determine whether the maladaptation our models predict actually corresponds to observed demographic or phenotypic responses in the field.

These considerations become more important when offset predictions are used to guide active interventions like assisted gene flow. In principle, moving individuals from donor populations that are pre‐adapted to future conditions can aid the evolutionary potential of vulnerable populations, but in practice the outcomes depend heavily on the quality of the genomic information guiding the intervention. Poorly informed translocations can result in real risks, including outbreeding depression and loss of locally adapted distinct genetic variation. The Chinese giant salamander ( *Andrias davidianus* ) is a well‐known example, where large‐scale indiscriminate restocking mixed once genetically and geographically distinct lineages (Yan et al. 2018). Furthermore, reintroducing ibex ( *Capra ibex* ) into the Tatra Mountains led to outbreeding depression and population collapse when hybrid offspring were born at unfavourable times of year because of using individuals sourced from climatically and ecologically mismatched populations (Frankham et al. 2011). In contrast, the Florida panther ( *Puma concolor coryi* ) genetic rescue plan demonstrates that genomically informed translocations can restore genetic diversity and fitness in an inbred population (Johnson et al. 2010). These different outcomes demonstrate that a successful assisted gene flow relies on matching donors to recipients using well‐supported genomic and ecological data. Because our results show geographic structure and lineage‐specific demographic histories in *D. grantii*, any assisted gene flow informed by these offset predictions should proceed cautiously, prioritizing the preservation of distinct lineages and validated donor–recipient matches over broad‐scale mixing.

Our analyses also showed that some populations may be relatively resilient to future climate change, whereas others may require active management attention to maintain or augment their evolutionary potential, particularly where projected environmental change may outpace existing adaptive genetic variation. In particular, our results provide a fine example of how genomic approaches can help meet the goals of the Kunming‐Montreal Global Biodiversity Framework by incorporating genetic indicators into biodiversity monitoring and supporting strategies for the long‐term sustainability of biodiversity resources (Hébert et al. 2026). Specifically, our results suggest several management priorities: (1) maintaining the extent and connectivity of Mogollon Rim montane forests as a reservoir of standing genetic variation, (2) targeted demographic and genetic monitoring of small, isolated, high‐inbreeding populations such as Utah, (3) integrating genomic indicators of decline, inbreeding and maladaptation into long‐term monitoring of the sky island archipelago, and (4) approaching any assisted gene flow cautiously, prioritizing validated donor–recipient matches over broad‐scale mixing.

Here, we document three key patterns: fragmentation‐driven drift, signal of polygenic adaptation to monsoon precipitation, and a strong role of historical connectivity in shaping standing genetic variation. These patterns raise broader questions about how the same evolutionary forces scale across the genus *Dynastes*. *Dynastes grantii* sits at the northern edge of the genus, whereas most *Dynastes* diversity is concentrated in the montane forests of Central and South America (Dutrillaux and Dutrillaux 2013; Morón 2009), where multiple species occupy similarly fragmented but topographically and climatically far more complex mountain systems. Comparative genomic work spanning the full latitudinal range of the genus is now needed to test whether the signature of Last Glacial Period expansion and Holocene contraction we recover here is phylogenetically conserved across *Dynastes*, and whether monsoon seasonality remains the dominant selective axis in tropical congeners, where rainfall is less strongly seasonal. Such work would offer a rare opportunity to separate the contingent history of a single peripheral population from the general principles governing how montane beetle lineages diversify across topographically fragmented landscapes (Mitchell and Ober 2013; Smith and Farrell 2005). At deeper divergence times, these same processes of isolation and drift may also underlie the origin of species boundaries within the genus. More broadly, the southwestern sky islands have served as both Pleistocene refugia and Holocene crucibles of divergence for cool‐adapted lineages (Betancourt et al. 1990; McCormack et al. 2008; Wiens et al. 2019), and the demographic and adaptive signatures preserved in their resident faunas provide a rare window into how montane biotas have repeatedly assembled, fragmented, and reassembled in response to climate oscillations (Love et al. 2023). Sustaining this evolutionary legacy into the Anthropocene will require integrating genomic indicators of demographic decline, inbreeding and maladaptation into long‐term biodiversity monitoring and management of the sky island archipelago (Hébert et al. 2026), so that the same processes that generated this diversity are not, under accelerating climate change, the ones that erase it.

## Author Contributions

Data curation and acquisition: S.C, M.‐H.L., J.‐P.H. (S.C., J.‐P.H. collected samples; S.C., M.‐H.L. conducted DNA extraction) Writing: S.C., H.B., J.‐P.H. (S.C., J.‐P.H. drafted the manuscript; S.C., H.B., J.‐P.H. edited and revised the manuscript).

## Funding

This work was supported by National Institute of General Medical Sciences at the National Institutes of Health R35GM138098, Society of Systematic Biologists Graduate Student Research Award and Ministry of Science and Technology of Taiwan (MOST 108‐2621‐B‐001‐001‐MY3).

## Ethics Statement

The authors have nothing to report.

## Conflicts of Interest

The authors declare no conflicts of interest.

## Supporting information


**Figure S1:** Distribution of sequencing depth and missing data across *D. grantii* samples. (A) Mean sequencing depth per individual. (B) Mean depth per site. (C) Missing data per individual (% of sites with no genotype call). (D) Missing data per site (% of individuals with missing genotype at a given SNP).
**Figure S2:** Scree plot and PCA of *D. grantii* genomic variation. (A) Scree plot shows the cumulative variance explained by the first 20 PCs. PC1‐PC3 explain 21.1% of total genomic variance, with PC1 explaining 9.9%, PC2 explaining 6.5% and PC3 explaining 5.7%. (B) PC1 vs. PC2 shows clear separation among major geographic lineages. PC1 primarily separates the Utah population from all southern populations. PC2 further differentiates the Mogollon Rim from the sky island system. (C) PC1 vs. PC3 shows the separation between Mt. Lemmon and other southern sky island populations. (D) PC2 vs. PC3 shows fine‐scale structure among the Mogollon Rim, sky island and peripheral populations.
**Figure S3:** Demographic history of *D. grantii* using SMC++. (A) Generation time 1 year mutation rate 3.0 × 10^−9^. (B) Generation time 1 year mutation rate 5.8 × 10^−9^. (C) Generation time 2 years mutation rate 3.0 × 10^−9^. (D) Generation time 2 years mutation rate 5.8 × 10^−9^.
**Figure S4:** Distribution of runs of homozygosity (ROH) segment lengths across populations. Histograms showing the frequency distribution of ROH segment lengths (in megabases, log‐scale) for (A) Mt. Lemmon, (B) Chiricahua Mt., (C) Mogollon Rim, and (D) Utah populations. All populations have ROH segments predominantly concentrated in the short length class (< 0.1 Mb) without long ROH segments (> 5 Mb).
**Table S1:** Sample information and sequencing metadata. Sample ID corresponds to the identifier used throughout the manuscript, figures and analyses. This table lists all individuals sequenced and submitted to the NCBI Sequence Read Archive (SRA). Samples failing quality‐control thresholds were excluded from downstream analyses. Locality information reflects the field collection site for newly collected specimens or the specimen label data for museum/private collections. For specimens lacking precise GPS coordinates (e.g., Payson and Reserve on the Mogollon Rim), a representative coordinate (Payson, AZ [34.25504–111.25847], Reserve, AZ [33.70516385–108.756497]) for the named locality was used in analyses requiring geographic position.
**Table S2:** ADMIXTURE cross‐validation (CV) error for *K* = 2–9.
**Table S3:** Demographic model comparison and parameter estimates. Results from δaδi 3D‐SFS demographic modelling comparing isolation with size change (ISC) models to simple isolation (SI) models. All population pairs show ΔAIC values ≤ 0.45, suggesting the data are consistent with simple isolation models.
**Table S4:** Genome‐wide (5 kb‐window) *D*‐statistic (ABBA‐BABA) test results. *D*‐statistic tests and compares all population trios to detect signatures of historical admixture. P1, P2 and P3 represent the three focal populations. *p*‐value indicates statistical significance (asterisks denote significant results at *α* < 0.05). Significant *D* values indicate deviation from the no‐admixture null hypothesis.
**Table S5:** Genome‐wide (5 kb‐window) *D*‐statistic (ABBA‐BABA) test results (Exclude a historical sample from Cochise).
**Table S6:** Genetic offset values for each population across climate scenarios. Genetic offset is shown for each population under six climate scenarios combining two emission pathways (SSP2‐4.5; SSP5‐8.5) and three future time windows (2041–2060, 2061–2080, 2081–2100). Mt. Lemmon emerges as the most vulnerable, with offset values substantially higher than the other three populations across all scenarios. The three remaining populations show similar vulnerability levels with notably lower offset values, suggesting they are better adapted to withstand projected climate changes.

## Data Availability

Raw sequence reads are deposited in the SRA (BioProject PRJNA1473909). Analysis scripts are available in the GitHub repository (https://github.com/seanchien4/Dynastes_grantii_popgen).

## References

[mec70551-bib-0001] Alexander, D. H. , J. Novembre , and K. Lange . 2009. “Fast Model‐Based Estimation of Ancestry in Unrelated Individuals.” Genome Research 19, no. 9: 1655–1664. 10.1101/gr.094052.109 PMC2752134 19648217

[mec70551-bib-0002] Betancourt, J. L. , T. R. Van Devender , and P. S. Martin . 1990. PACKRAT MIDDENS: The Last 40,000 Years of Biotic Change. University of Arizona Press. 10.1126/science.250.4983.1021-a 17746928

[mec70551-bib-0003] Brůna, T. , K. J. Hoff , A. Lomsadze , M. Stanke , and M. Borodovsky . 2021. “BRAKER2: Automatic Eukaryotic Genome Annotation With GeneMark‐EP+ and AUGUSTUS Supported by a Protein Database.” NAR Genomics and Bioinformatics 3, no. 1: lqaa108. 10.1093/nargab/lqaa108 PMC7787252 33575650

[mec70551-bib-0004] Brůna, T. , A. Lomsadze , and M. Borodovsky . 2020. “GeneMark‐EP+: Eukaryotic Gene Prediction With Self‐Training in the Space of Genes and Proteins.” NAR Genomics and Bioinformatics 2, no. 2: lqaa026. 10.1093/nargab/lqaa026 PMC7222226 32440658

[mec70551-bib-0005] Buchfink, B. , C. Xie , and D. H. Huson . 2015. “Fast and Sensitive Protein Alignment Using DIAMOND.” Nature Methods 12, no. 1: 59–60. 10.1038/nmeth.3176 25402007

[mec70551-bib-0006] Capblancq, T. , and B. R. Forester . 2021. “Redundancy Analysis: A Swiss Army Knife for Landscape Genomics.” Methods in Ecology and Evolution 12, no. 12: 2298–2309.

[mec70551-bib-0007] Caye, K. , B. Jumentier , J. Lepeule , and O. François . 2019. “LFMM 2: Fast and Accurate Inference of Gene‐Environment Associations in Genome‐Wide Studies.” Molecular Biology and Evolution 36, no. 4: 852–860. 10.1093/molbev/msz008 PMC6659841 30657943

[mec70551-bib-0009] Danecek, P. , A. Auton , G. Abecasis , et al. 2011. “The Variant Call Format and VCFtools.” Bioinformatics 27, no. 15: 2156–2158. 10.1093/bioinformatics/btr330 PMC3137218 21653522

[mec70551-bib-0010] Derkarabetian, S. , M. Burns , J. Starrett , and M. Hedin . 2016. “Population Genomic Evidence for Multiple Pliocene Refugia in a Montane‐Restricted Harvestman (Arachnida, Opiliones, *Sclerobunus robustus* ) From the Southwestern United States.” Molecular Ecology 25, no. 18: 4611–4631. 10.1111/mec.13789 27483047

[mec70551-bib-0011] Dixon, P. 2003. “VEGAN, a Package of R Functions for Community Ecology.” Journal of Vegetation Science 14, no. 6: 927–930.

[mec70551-bib-0012] Dutrillaux, B. , and A.‐M. Dutrillaux . 2013. “A South American Origin of the Genus Dynastes (Coleoptera: Scarabaeidae: Dynastinae) Demonstrated by Chromosomal Analyses.” Cytogenetic and Genome Research 141, no. 1: 37–42. 10.1159/000351210 23735513

[mec70551-bib-0013] Fick, S. E. , and R. J. Hijmans . 2017. “WorldClim 2: New 1‐km Spatial Resolution Climate Surfaces for Global Land Areas: New Climate Surfaces for Global Land Areas.” International Journal of Climatology: A Journal of the Royal Meteorological Society 37, no. 12: 4302–4315.

[mec70551-bib-0014] Fitzpatrick, M. C. , and S. R. Keller . 2015. “Ecological Genomics Meets Community‐Level Modelling of Biodiversity: Mapping the Genomic Landscape of Current and Future Environmental Adaptation.” Ecology Letters 18, no. 1: 1–16. 10.1111/ele.12376 25270536

[mec70551-bib-0015] Fleischner, T. L. , M. L. Floyd , J. Rack , et al. 2024. “The Mogollon Highlands Ecoregion of the American Southwest: A Neglected Center of Ecological Diversity.” Natural Areas Journal 44, no. 2: 104–119.

[mec70551-bib-0016] Forester, B. R. , J. R. Lasky , H. H. Wagner , and D. L. Urban . 2018. “Comparing Methods for Detecting Multilocus Adaptation With Multivariate Genotype‐Environment Associations.” Molecular Ecology 27, no. 9: 2215–2233. 10.1111/mec.14584 29633402

[mec70551-bib-0017] Frankham, R. , J. D. Ballou , M. D. B. Eldridge , et al. 2011. “Predicting the Probability of Outbreeding Depression: Predicting Outbreeding Depression.” Conservation Biology: The Journal of the Society for Conservation Biology 25, no. 3: 465–475. 10.1111/j.1523-1739.2011.01662.x 21486369

[mec70551-bib-0018] Frey, J. K. 2026. “Biogeography of Montane Mammals in the Great Basin and American Southwest: Review and Perspectives for the Future.” Journal of Mammalogy: gyag033. 10.1093/jmammal/gyag033.

[mec70551-bib-0019] Gabriel, L. , K. J. Hoff , T. Brůna , M. Borodovsky , and M. Stanke . 2021. “TSEBRA: Transcript Selector for BRAKER.” BMC Bioinformatics 22, no. 1: 566. 10.1186/s12859-021-04482-0 PMC8620231 34823473

[mec70551-bib-0020] Gain, C. , B. Rhoné , P. Cubry , et al. 2023. “A Quantitative Theory for Genomic Offset Statistics.” Molecular Biology and Evolution 40, no. 6: msad140. 10.1093/molbev/msad140 PMC10306404 37307566

[mec70551-bib-0021] Garg, K. M. , B. Chattopadhyay , B. Koane , K. Sam , and F. E. Rheindt . 2020. “Last Glacial Maximum Led to Community‐Wide Population Expansion in a Montane Songbird Radiation in Highland Papua New Guinea.” BMC Evolutionary Biology 20, no. 1: 82. 10.1186/s12862-020-01646-z PMC7353695 32652951

[mec70551-bib-0022] GBIF.org User . 2026. Occurrence Download [Dataset]. Global Biodiversity Information Facility. 10.15468/DL.VRC9DS.

[mec70551-bib-0023] Gotoh, O. 2008. “A Space‐Efficient and Accurate Method for Mapping and Aligning cDNA Sequences Onto Genomic Sequence.” Nucleic Acids Research 36, no. 8: 2630–2638. 10.1093/nar/gkn105 PMC2377433 18344523

[mec70551-bib-0024] Gutenkunst, R. N. , R. D. Hernandez , S. H. Williamson , and C. D. Bustamante . 2009. “Inferring the Joint Demographic History of Multiple Populations From Multidimensional SNP Frequency Data.” PLoS Genetics 5, no. 10: e1000695. 10.1371/journal.pgen.1000695 PMC2760211 19851460

[mec70551-bib-0025] Haire, S. L. , M. L. Villarreal , C. Cortés‐Montaño , et al. 2022. “Climate Refugia for Pinus spp. in Topographic and Bioclimatic Environments of the Madrean Sky Islands of México and the United States.” Plant Ecology 223, no. 5: 577–598.

[mec70551-bib-0026] Halbritter, D. A. , C. G. Storer , A. Y. Kawahara , and J. C. Daniels . 2019. “Phylogeography and Population Genetics of Pine Butterflies: Sky Islands Increase Genetic Divergence.” Ecology and Evolution 9, no. 23: 13389–13401. 10.1002/ece3.5793 PMC6912906 31871652

[mec70551-bib-0027] Hébert, K. , L. Pollock , and S. Hoban . 2026. “How Much Monitoring Is Needed to Reliably Track Progress Towards Genetic Diversity Targets?” Biological Conservation 317: 111824.

[mec70551-bib-0028] Hoff, K. J. , S. Lange , A. Lomsadze , M. Borodovsky , and M. Stanke . 2016. “BRAKER1: Unsupervised RNA‐Seq‐Based Genome Annotation With GeneMark‐ET and AUGUSTUS.” Bioinformatics 32, no. 5: 767–769. 10.1093/bioinformatics/btv661 PMC6078167 26559507

[mec70551-bib-0030] Huang, J.‐P. 2019. “Holocene Population Decline and Conservation Implication for the Western Hercules Beetle, *Dynastes grantii* (Coleoptera, Scarabaeidae).” Journal of Heredity 110, no. 5: 629–637. 10.1093/jhered/esz036 31135893

[mec70551-bib-0031] Iwata, H. , and O. Gotoh . 2012. “Benchmarking Spliced Alignment Programs Including Spaln2, an Extended Version of Spaln That Incorporates Additional Species‐Specific Features.” Nucleic Acids Research 40, no. 20: e161. 10.1093/nar/gks708 PMC3488211 22848105

[mec70551-bib-0032] Johnson, W. E. , D. P. Onorato , M. E. Roelke , et al. 2010. “Genetic Restoration of the Florida Panther.” Science 329, no. 5999: 1641–1645. 10.1126/science.1192891 PMC6993177 20929847

[mec70551-bib-0033] Jung, Y. , and D. Han . 2022. “BWA‐MEME: BWA‐MEM Emulated With a Machine Learning Approach.” Bioinformatics 38, no. 9: 2404–2413. 10.1093/bioinformatics/btac137 35253835

[mec70551-bib-0034] Keightley, P. D. , R. W. Ness , D. L. Halligan , and P. R. Haddrill . 2014. “Estimation of the Spontaneous Mutation Rate Per Nucleotide Site in a *Drosophila melanogaster* Full‐Sib Family.” Genetics 196, no. 1: 313–320. 10.1534/genetics.113.158758 PMC3872194 24214343

[mec70551-bib-0035] Korunes, K. L. , and K. Samuk . 2021. “Pixy: Unbiased Estimation of Nucleotide Diversity and Divergence in the Presence of Missing Data.” Molecular Ecology Resources 21, no. 4: 1359–1368. 10.1111/1755-0998.13326 PMC8044049 33453139

[mec70551-bib-0036] Kuznetsov, D. , F. Tegenfeldt , M. Manni , et al. 2023. “OrthoDB v11: Annotation of Orthologs in the Widest Sampling of Organismal Diversity.” Nucleic Acids Research 51, no. D1: D445–D451. 10.1093/nar/gkac998 PMC9825584 36350662

[mec70551-bib-0037] Láruson, Á. J. , M. C. Fitzpatrick , S. R. Keller , B. C. Haller , and K. E. Lotterhos . 2022. “Seeing the Forest for the Trees: Assessing Genetic Offset Predictions From Gradient Forest.” Evolutionary Applications 15, no. 3: 403–416. 10.1111/eva.13354 PMC8965365 35386401

[mec70551-bib-0038] Lawson, D. J. , L. van Dorp , and D. Falush . 2018. “A Tutorial on How Not to Over‐Interpret STRUCTURE and ADMIXTURE Bar Plots.” Nature Communications 9, no. 1: 3258. 10.1038/s41467-018-05257-7 PMC6092366 30108219

[mec70551-bib-0039] Li, H. , B. Handsaker , A. Wysoker , et al. 2009. “The Sequence Alignment/Map Format and SAMtools.” Bioinformatics 25, no. 16: 2078–2079. 10.1093/bioinformatics/btp352 PMC2723002 19505943

[mec70551-bib-0040] Lomsadze, A. , V. Ter‐Hovhannisyan , Y. O. Chernoff , and M. Borodovsky . 2005. “Gene Identification in Novel Eukaryotic Genomes by Self‐Training Algorithm.” Nucleic Acids Research 33, no. 20: 6494–6506. 10.1093/nar/gki937 PMC1298918 16314312

[mec70551-bib-0041] Love, S. J. , J. A. Schweitzer , S. A. Woolbright , and J. K. Bailey . 2023. “Sky Islands Are a Global Tool for Predicting the Ecological and Evolutionary Consequences of Climate Change.” Annual Review of Ecology, Evolution, and Systematics 54, no. 1: 219–236.

[mec70551-bib-0042] Malinsky, M. , M. Matschiner , and H. Svardal . 2021. “Dsuite ‐ Fast D‐Statistics and Related Admixture Evidence From VCF Files.” Molecular Ecology Resources 21, no. 2: 584–595. 10.1111/1755-0998.13265 PMC7116594 33012121

[mec70551-bib-0043] Masta, S. E. 2000. “Phylogeography of the Jumping Spider *Habronattus pugillis* (Araneae: Salticidae): Recent Vicariance of Sky Island Populations?” Evolution 54, no. 5: 1699–1711. 10.1111/j.0014-3820.2000.tb00714.x 11108597

[mec70551-bib-0044] McCormack, J. E. , B. S. Bowen , and T. B. Smith . 2008. “Integrating Paleoecology and Genetics of Bird Populations in Two Sky Island Archipelagos.” BMC Biology 6, no. 1: 28. 10.1186/1741-7007-6-28 PMC2474579 18588695

[mec70551-bib-0045] McKenna, A. , M. Hanna , E. Banks , et al. 2010. “The Genome Analysis Toolkit: A MapReduce Framework for Analyzing Next‐Generation DNA Sequencing Data.” Genome Research 20, no. 9: 1297–1303. 10.1101/gr.107524.110 PMC2928508 20644199

[mec70551-bib-0046] McMonigle, O. 2012. The Ultimate Guide to Breeding Beetles: Coleoptera Laboratory Culture Methods. Coachwhip Publications.

[mec70551-bib-0047] Menke, A. S. , and F. D. Parker . 1988. “Adult Feeding and Distribution of *Dynastes granti* Horn (Coleoptera: Scarabaeidae).” Coleopterists Bulletin 42, no. 2: 161–164.

[mec70551-bib-0048] Meyer, W. M., 3rd , J. A. Eble , K. Franklin , et al. 2015. “Ground‐Dwelling Arthropod Communities of a Sky Island Mountain Range in Southeastern Arizona, USA: Obtaining a Baseline for Assessing the Effects of Climate Change.” PLoS One 10, no. 9: e0135210. 10.1371/journal.pone.0135210 PMC4558002 26332685

[mec70551-bib-0049] Miles, A. , N. J. Harding , G. Bottà , et al. 2017. “Genetic Diversity of the African Malaria Vector *Anopheles gambiae* .” Nature 552, no. 7683: 96–100. 10.1038/nature24995 PMC6026373 29186111

[mec70551-bib-0050] Mitchell, S. G. , and K. A. Ober . 2013. “Evolution of *Scaphinotus petersi* (Coleoptera: Carabidae) and the Role of Climate and Geography in the Madrean Sky Islands of Southeastern Arizona, USA.” Quaternary Research 79, no. 2: 274–283.

[mec70551-bib-0051] Moore, W. , W. M. Meyer III , J. A. Eble , K. Franklin , J. F. Wiens , and R. C. Brusca . 2013. “Introduction to the Arizona Sky Island Arthropod Project (ASAP): Systematics, biogeography, ecology, and population genetics of arthropods of the Madrean Sky Islands.” In Merging science and management in a rapidly changing world: Biodiversity and management of the Madrean Archipelago III and 7th Conference on Research and Resource Management in the Southwestern Deserts, edited by G. J. Gottfried , P. F. Ffolliott , B. S. Gebow , L. G. Eskew , and L. C. Collins , 144–168. U.S. Department of Agriculture. PMC4260471 25505938

[mec70551-bib-0052] Morón, M. 2009. “El Género Dynastes Mac Leay,1819 en la zona de Transición Mexicana (Coleoptera:Melolonthidae: Dynastinae).” Boletín de la SEA 45: 23–38.

[mec70551-bib-0053] Naimi, B. , N. A. S. Hamm , T. A. Groen , A. K. Skidmore , and A. G. Toxopeus . 2014. “Where Is Positional Uncertainty a Problem for Species Distribution Modelling?” Ecography 37, no. 2: 191–203.

[mec70551-bib-0054] Purcell, S. , B. Neale , K. Todd‐Brown , et al. 2007. “PLINK: A Tool Set for Whole‐Genome Association and Population‐Based Linkage Analyses.” American Journal of Human Genetics 81, no. 3: 559–575. 10.1086/519795 PMC1950838 17701901

[mec70551-bib-0055] Ratcliffe, B. C. , and R. D. Cave . 2017. The Dynastine Scarab Beetles of the USA and Canada (Coleoptera: Scarabaeidae: Dynastinae) (Vol. 30). University of Nebraska.

[mec70551-bib-0056] Ratcliffe, B. C. , R. D. Cave , and E. B. Cano . 2013. The Dynastine Scarab Beetles of Mexico, Guatemala, and Belize (Coleoptera: Scarabaeidae: Dynastinae) (Vol. 27). University of Nebraska.

[mec70551-bib-0057] Rellstab, C. , B. Dauphin , and M. Exposito‐Alonso . 2021. “Prospects and Limitations of Genomic Offset in Conservation Management.” Evolutionary Applications 14, no. 5: 1202–1212. 10.1111/eva.13205 PMC8127717 34025760

[mec70551-bib-0058] Rumberger, C. A. , M. G. Eppley , K. Bajaj , et al. 2026. “Genomic Offsets Predict Survival With Low Accuracy in a Marine Common Garden.” Molecular Ecology 35, no. 13: e70457. 10.1111/mec.70457 PMC13332419 42400347

[mec70551-bib-0059] Santiago, E. , C. Köpke , and A. Caballero . 2025. “Accounting for Population Structure and Data Quality in Demographic Inference With Linkage Disequilibrium Methods.” Nature Communications 16, no. 1: 6054. 10.1038/s41467-025-61378-w PMC12218012 40592851

[mec70551-bib-0060] Smith, C. I. , and B. D. Farrell . 2005. “Phylogeography of the Longhorn Cactus Beetle *Moneilema appressum* LeConte (Coleoptera: Cerambycidae): Was the Differentiation of the Madrean Sky Islands Driven by Pleistocene Climate Changes?” Molecular Ecology 14, no. 10: 3049–3065. 10.1111/j.1365-294X.2005.02647.x 16101773

[mec70551-bib-0061] Stanke, M. , M. Diekhans , R. Baertsch , and D. Haussler . 2008. “Using Native and Syntenically Mapped cDNA Alignments to Improve de Novo Gene Finding.” Bioinformatics 24, no. 5: 637–644. 10.1093/bioinformatics/btn013 18218656

[mec70551-bib-0062] Stanke, M. , O. Schöffmann , B. Morgenstern , and S. Waack . 2006. “Gene Prediction in Eukaryotes With a Generalized Hidden Markov Model That Uses Hints From External Sources.” BMC Bioinformatics 7, no. 1: 62. 10.1186/1471-2105-7-62 PMC1409804 16469098

[mec70551-bib-0063] Terhorst, J. , J. A. Kamm , and Y. S. Song . 2017. “Robust and Scalable Inference of Population History From Hundreds of Unphased Whole Genomes.” Nature Genetics 49, no. 2: 303–309. 10.1038/ng.3748 PMC5470542 28024154

[mec70551-bib-0064] Warshall, P. 1995. Biodiversity and Management of the Madrean Archipelago: The Sky Islands of Southwestern United States and Northwestern Mexico. US Department of Agriculture, Forest Service.

[mec70551-bib-0065] Wiens, J. J. , A. Camacho , A. Goldberg , et al. 2019. “Climate Change, Extinction, and Sky Island Biogeography in a Montane Lizard.” Molecular Ecology 28, no. 10: 2610–2624. 10.1111/mec.15073 30843297

[mec70551-bib-0066] Wilfert, L. , J. Gadau , and P. Schmid‐Hempel . 2007. “Variation in Genomic Recombination Rates Among Animal Taxa and the Case of Social Insects.” Heredity 98, no. 4: 189–197. 10.1038/sj.hdy.6800950 17389895

[mec70551-bib-0067] Xu, S. , S. Al‐Madhagy , P. Duchen , and A. Edison . 2026. “Trio‐Sequencing Reveals High Germline Mutation Rates in the Colorado Potato Beetle ( *Leptinotarsa decemlineata* ).” Genome Biology and Evolution 18, no. 2: evag027. 10.1093/gbe/evag027 PMC12914578 41635933

[mec70551-bib-0068] Yan, F. , J. Lü , B. Zhang , et al. 2018. “The Chinese Giant Salamander Exemplifies the Hidden Extinction of Cryptic Species.” Current Biology 28, no. 10: R590–R592. 10.1016/j.cub.2018.04.004 29787716

[mec70551-bib-0069] Zdobnov, E. M. , and R. Apweiler . 2001. “InterProScan—An Integration Platform for the Signature‐Recognition Methods in InterPro.” Bioinformatics 17, no. 9: 847–848. 10.1093/bioinformatics/17.9.847 11590104

