# Supplementary material for: Differential Demographic Trajectories and Polygenic Adaptation to Monsoon Seasonality in Sky Island Hercules Beetle Populations of the American Southwest
Source: Mol Ecol. 2026 Sep 25;35(18):e70551. doi: 10.1111/mec.70551 (PMC13615329; doi:10.1111/mec.70551)
Supplement: Supplementary file 1 [file MEC-35-e70551-s001.docx]

### **Supplementary**

**Figure S1. Distribution of sequencing depth and missing data across *D. grantii* samples.** A) Mean sequencing depth per individual ranges. B) Mean depth per site. C) Missing data per individual (% of sites with no genotype call). D) Missing data per site (% of individuals with missing genotype at a given SNP).

**Figure S2. Scree plot and PCA of *D. grantii* genomic variation.** A) Scree plot shows the cumulative variance explained by the first 20 PCs. PC1-PC3 explain 21.1% of total genomic variance, with PC1 explaining 9.9%, PC2 explaining 6.5%, and PC3 explaining 5.7%. B) PC1 vs. PC2 shows clear separation among major geographic lineages. PC1 primarily separates the Utah population from all southern populations. PC2 further differentiates the Mogollon Rim from the sky island system. C) PC1 vs. PC3 shows the separation between Mt. Lemmon and other southern sky island populations. D) PC2 vs. PC3 shows fine-scale structure among the Mogollon Rim, sky island, and peripheral populations.

**Figure S3. Demographic history of *D. grantii* using SMC++.** A) Generation time 1 year mutation rate 3.0 × 10^-9^. B) Generation time 1 year mutation rate 5.8 × 10^-9^. C) Generation time 2 years mutation rate 3.0 × 10^-9^. D) Generation time 2 years mutation rate 5.8 ×10^-9^.

**Figure S4. Distribution of runs of homozygosity (ROH) segment lengths across populations.** Histograms showing the frequency distribution of ROH segment lengths (in megabases, log-scale) for A) Mt. Lemmon, B) Chiricahua Mt., C) Mogollon Rim, and D) Utah populations. All populations have ROH segments predominantly concentrated in the short length class (< 0.1 Mb) without long ROH segments (> 5 Mb).

| **ID** | **Location** | **Date** | **Lat** | **Lon** | **Repository** | **Biosample accession** |
| --- | --- | --- | --- | --- | --- | --- |
| C | Cochise County, AZ, USA | 8/1968 | N/A | N/A | Field Museum | SRR38956827 |
| P1 | Payson, AZ, USA | 2012 | N/A | N/A | MSB^1^ | SRR38956847 |
| P2 | Payson, AZ, USA | 2012 | N/A | N/A | MSB^1^ | SRR38956845 |
| P3 | Payson, AZ, USA | 2012 | N/A | N/A | MSB^1^ | SRR38956844 |
| P4 | Payson, AZ, USA | 2012 | N/A | N/A | MSB^1^ | SRR38956843 |
| P5 | Payson, AZ, USA | 2012 | N/A | N/A | MSB^1^ | SRR38956842 |
| P6 | Payson, AZ, USA | 2012 | N/A | N/A | MSB^1^ | SRR38956841 |
| R1 | Reserve, NM, USA | 2012 | N/A | N/A | MSB^1^ | SRR38956840 |
| R2 | Reserve, NM, USA | 2012 | N/A | N/A | MSB^1^ | SRR38956839 |
| R3 | Reserve, NM, USA | 2012 | N/A | N/A | MSB^1^ | SRR38956838 |
| R4 | Reserve, NM, USA | 2012 | N/A | N/A | MSB^1^ | SRR38956837 |
| R5 | Reserve, NM, USA | 2012 | N/A | N/A | MSB^1^ | SRR38956836 |
| R6 | Reserve, NM, USA | 2012 | N/A | N/A | MSB^1^ | SRR38956834 |
| UN1 | Unknown^2^ | 10/2019 | N/A | N/A | BRMAS^3^ | SRR38956833 |
| UN2 | Unknown^2^ | 10/2019 | N/A | N/A | BRMAS^3^ | SRR38956832 |
| UN3 | Unknown^2^ | 10/2019 | N/A | N/A | BRMAS^3^ | SRR38956831 |
| UN4 | Unknown^2^ | 10/2019 | N/A | N/A | BRMAS^3^ | SRR38956830 |
| UN5 | Unknown^2^ | 10/2019 | N/A | N/A | BRMAS^3^ | SRR38956829 |
| UN6 | Unknown^2^ | 10/2019 | N/A | N/A | BRMAS^3^ | SRR38956828 |
| UT1 | Kane County, UT, USA | 9/2019 | 37.222528 | -112.68175 | BRMAS^3^ | SRR38956852 |
| UT2 | Kane County, UT, USA | 9/2019 | 37.222528 | -112.68175 | BRMAS^3^ | SRR38956851 |
| UT4 | Kane County, UT, USA | 9/2019 | 37.222528 | -112.68175 | BRMAS^3^ | SRR38956850 |
| UT5 | Kane County, UT, USA | 9/2019 | 37.222528 | -112.68175 | BRMAS^3^ | SRR38956849 |
| UT6 | Kane County, UT, USA | 9/2019 | 37.222528 | -112.68175 | BRMAS^3^ | SRR38956848 |
| M | Bacanora, Sonora, Mexico | 10/18/2021 | N/A | N/A | BRMAS^3^ | SRR38956853 |
| MC1 | Mt. Wrighton | 9/5/2023 | 31.71387 | -110.87374 | TAMU^4^ | SRR38956858 |
| MC2 | Mt. Wrighton | 9/5/2023 | 31.71387 | -110.87374 | TAMU^4^ | SRR38956857 |
| MG1 | Mt. Graham | 8/31/2024 | 32.644867 | -109.81702 | TAMU^4^ | SRR38956846 |
| ML2 | Mt. Lemmoon | 9/6/2023 | 32.3746 | -110.691 | TAMU^4^ | SRR38956835 |
| ML3 | Mt. Lemmoon | 9/7/2023 | 32.3733 | -110.69176 | TAMU^4^ | SRR38956826 |
| ML4 | Mt. Lemmoon | 8/28/2024 | 32.37261 | -110.69415 | TAMU^4^ | SRR38956825 |
| ML5 | Mt. Lemmoon | 8/28/2024 | 32.37261 | -110.69415 | TAMU^4^ | SRR38956824 |
| ML6 | Mt. Lemmoon | 8/28/2024 | 32.37261 | -110.69415 | TAMU^4^ | SRR38956823 |
| PT1 | Chiricahua Mt. | 9/3/2023 | 31.8978 | -109.2173 | TAMU^4^ | SRR38956822 |
| PT2 | Chiricahua Mt. | 9/4/2023 | 31.8937 | -109.2147 | TAMU^4^ | SRR38956821 |
| PT3 | Chiricahua Mt. | 9/4/2023 | 31.8937 | -109.2147 | TAMU^4^ | SRR38956856 |
| PT4 | Chiricahua Mt. | 9/4/2023 | 31.8937 | -109.2147 | TAMU^4^ | SRR38956855 |
| PT5 | Chiricahua Mt. | 8/25/2024 | 31.89606 | -109.21618 | TAMU^4^ | SRR38956854 |

^1^Museum of Southwestern Biology, University of New Mexico

^2^Private collection

^3^Biodiversity Research Museum at Academia Sinica, Taiwan

^4^Texas A&M University

**Table S1. Sample information and sequencing metadata**. Sample ID corresponds to the identifier used throughout the manuscript, figures, and analyses. This table lists all individuals sequenced and submitted to the NCBI Sequence Read Archive (SRA). Samples failing quality-control thresholds were excluded from downstream analyses. Locality information reflects the field collection site for newly collected specimens or the specimen label data for museum/private collections. For specimens lacking precise GPS coordinates (e.g., Payson and Reserve on the Mogollon Rim), a representative coordinate (Payson, AZ [34.25504 -111.25847], Reserve, AZ [33.70516385 -108.756497]) for the named locality was used in analyses requiring geographic position.

| K | CV error |
| --- | --- |
| 2 | 0.79059 |
| 3 | 0.86325 |
| 4 | 1.04660 |
| 5 | 1.20852 |
| 6 | 1.43736 |
| 7 | 1.66904 |
| 8 | 1.97873 |
| 9 | 2.22857 |

**Table S2. ADMIXTURE cross-validation (CV) error for K = 2-9.**

| Pair | SI LL | ISC LL | ΔAIC | nu1 | nu2 | T_split |
| --- | --- | --- | --- | --- | --- | --- |
| Utah-Mogollon | -77.38 | -77.30 | 0.18 | 0.082176 | 50.600301 | 0.030239 |
| Chiricahua-Mogollon | -84.91 | -84.89 | 0.03 | 0.419840 | 3.308846 | 0.027598 |
| Lemmon-Mogollon | -83.44 | -83.43 | 0.02 | 2.901890 | 7.838110 | 0.050775 |
| Lemmon-Utah | -52.31 | -52.08 | 0.45 | 12.973941 | 0.177100 | 0.079343 |
| Lemmon-Chiricahua | -56.96 | -56.95 | 0.02 | 8.884187 | 0.332549 | 0.024317 |
| Chiricahua-Utah | -49.28 | -49.16 | 0.23 | 10.275965 | 0.279237 | 0.134266 |

**Table S3. Demographic model comparison and parameter estimates.** Results from δaδi 3D-SFS demographic modeling comparing isolation with size change(ISC) models to simple isolation (SI) models. All population pairs show ΔAIC values ≤ 0.45, suggesting the data are consistent with simple isolation models.

| P1 | P2 | P3 | *D* | *Z* | *p-value* | *f*_4_ |
| --- | --- | --- | --- | --- | --- | --- |
| Cochise | Graham | Chiricahua | 0.0009 | 0.1552 | 0.8767 | 0.0039 |
| Cochise | Lemmon | Chiricahua | 0.0138 | 3.7306* | 0.0002* | 0.0608 |
| Cochise | Chiricahua | Mexico | 0.0018 | 0.6317 | 0.5276 | 0.0029 |
| Cochise | Chiricahua | Mogollon | 0.0077 | 3.4966* | 0.0005* | 0.1156 |
| Chiricahua | Wrighton | Cochise | 0.0060 | 1.7480 | 0.0805 | 0.0089 |
| Chiricahua | Lemmon | Graham | 0.0041 | 1.7241 | 0.0847 | 0.0065 |
| Graham | Chiricahua | Mexico | 0.0053 | 1.7697 | 0.0768 | 0.0081 |
| Graham | Chiricahua | Mogollon | 0.0065 | 2.5354* | 0.0112* | 0.0993 |
| Wrighton | Chiricahua | Graham | 0.0028 | 0.9612 | 0.3365 | 0.0044 |
| Lemmon | Chiricahua | Mexico | 0.0004 | 0.2704 | 0.7868 | 0.0006 |
| Lemmon | Chiricahua | Mogollon | 0.0004 | 0.2982 | 0.7655 | 0.0065 |
| Lemmon | Chiricahua | Wrighton | 0.0033 | 1.4408 | 0.1496 | 0.0050 |
| Mogollon | Chiricahua | Mexico | 0.0004 | 0.2115 | 0.8325 | 0.0007 |
| Wrighton | Chiricahua | Mexico | 0.0018 | 0.4965 | 0.6196 | 0.0027 |
| Wrighton | Chiricahua | Mogollon | 0.0046 | 1.6515 | 0.0986 | 0.0710 |
| Cochise | Lemmon | Graham | 0.0176 | 3.9549* | 0.0001* | 0.0270 |
| Graham | Cochise | Mexico | 0.0035 | 0.9378 | 0.3483 | 0.0055 |
| Cochise | Graham | Mogollon | 0.0017 | 0.5200 | 0.6030 | 0.0259 |
| Graham | Cochise | Wrighton | 0.0080 | 1.3742 | 0.1694 | 0.0121 |
| Cochise | Lemmon | Mexico | 0.0011 | 0.3527 | 0.7243 | 0.0017 |
| Cochise | Lemmon | Mogollon | 0.0072 | 3.7132* | 0.0002* | 0.1057 |
| Cochise | Lemmon | Wrighton | 0.0129 | 2.7505* | 0.0059* | 0.0191 |
| Mogollon | Mexico | Cochise | 0.0003 | 0.0880 | 0.9299 | 0.0005 |
| Wrighton | Cochise | Mexico | 0.0003 | 0.0649 | 0.9483 | 0.0004 |
| Cochise | Wrighton | Mogollon | 0.0030 | 1.6527 | 0.0984 | 0.0450 |
| Graham | Lemmon | Mexico | 0.0051 | 1.6431 | 0.1004 | 0.0078 |
| Graham | Lemmon | Mogollon | 0.0066 | 2.4207* | 0.0155* | 0.0999 |
| Wrighton | Lemmon | Graham | 0.0069 | 2.2412* | 0.0250* | 0.0110 |
| Mexico | Graham | Mogollon | 0.0009 | 0.2060 | 0.8368 | 0.0135 |
| Graham | Wrighton | Mexico | 0.0035 | 0.6607 | 0.5088 | 0.0053 |
| Graham | Wrighton | Mogollon | 0.0020 | 0.5575 | 0.5772 | 0.0310 |
| Mogollon | Lemmon | Mexico | 0.0000 | 0.0232 | 0.9815 | 0.0001 |
| Wrighton | Lemmon | Mexico | 0.0017 | 0.4367 | 0.6624 | 0.0026 |
| Wrighton | Lemmon | Mogollon | 0.0042 | 1.5938 | 0.1110 | 0.0656 |
| Wrighton | Mogollon | Mexico | 0.0014 | 0.4003 | 0.6890 | 0.0022 |

**Table S4. Genome-wide** **(5kb-window) D-statistic (ABBA-BABA) test results.** D-statistic tests and compares all population trios to detect signatures of historical admixture. P1, P2, and P3 represent the three focal populations. *P-value* indicates statistical significance (asterisks denote significant results at α < 0.05). Significant *D* values indicate deviation from the no-admixture null hypothesis.

| P1 | P2 | P3 | *D* | *Z* | *p-value* | *f*_4_ |
| --- | --- | --- | --- | --- | --- | --- |
| Chiricahua | Lemmon | Graham | 0.0041 | 1.7241 | 0.0847 | 0.0065 |
| Graham | Chiricahua | Mexico | 0.0053 | 1.7697 | 0.0768 | 0.0081 |
| Graham | Chiricahua | Mogollon | 0.0065 | 2.5354* | 0.0112* | 0.0992 |
| Wrighton | Chiricahua | Graham | 0.0028 | 0.9612 | 0.3365 | 0.0044 |
| Lemmon | Chiricahua | Mexico | 0.0004 | 0.2704 | 0.7868 | 0.0006 |
| Lemmon | Chiricahua | Mogollon | 0.0004 | 0.2982 | 0.7655 | 0.0065 |
| Lemmon | Chiricahua | Wrighton | 0.0033 | 1.4408 | 0.1496 | 0.0050 |
| Mogollon | Chiricahua | Mexico | 0.0004 | 0.2115 | 0.8325 | 0.0007 |
| Wrighton | Chiricahua | Mexico | 0.0018 | 0.4965 | 0.6196 | 0.0027 |
| Wrighton | Chiricahua | Mogollon | 0.0046 | 1.6515 | 0.0986 | 0.0713 |
| Graham | Lemmon | Mexico | 0.0051 | 1.6431 | 0.1004 | 0.0078 |
| Graham | Lemmon | Mogollon | 0.0066 | 2.4207* | 0.0155* | 0.0997 |
| Wrighton | Lemmon | Graham | 0.0069 | 2.2412* | 0.0250* | 0.0110 |
| Mexico | Graham | Mogollon | 0.0009 | 0.2060 | 0.8368 | 0.0134 |
| Graham | Wrighton | Mexico | 0.0035 | 0.6607 | 0.5088 | 0.0053 |
| Graham | Wrighton | Mogollon | 0.0020 | 0.5575 | 0.5772 | 0.0310 |
| Mogollon | Lemmon | Mexico | 0.0000 | 0.0232 | 0.9815 | 0.0001 |
| Wrighton | Lemmon | Mexico | 0.0017 | 0.4367 | 0.6624 | 0.0026 |
| Wrighton | Lemmon | Mogollon | 0.0042 | 1.5938 | 0.1110 | 0.0659 |
| Wrighton | Mogollon | Mexico | 0.0014 | 0.4003 | 0.6890 | 0.0022 |

**Table S5. Genome-wide** **(5kb-window) D-statistic (ABBA-BABA) test results (Exclude a historical sample from Cochise).**

|  | 245_2041 | 585_2041 | 245_2061 | 585_2061 | 245_2081 | 585_2081 |
| --- | --- | --- | --- | --- | --- | --- |
| Mt. Lemmon | 1,015.2 | 1,055.5 | 1,036.6 | 994.1 | 1,022.0 | 929.2 |
| Chiricahua Mt. | 588.5 | 626.1 | 632.4 | 644.5 | 628.9 | 579.1 |
| Mogollon Rim | 660.0 | 674.4 | 669.3 | 619.6 | 642.3 | 608.5 |
| Utah | 591.8 | 667.8 | 681.6 | 594.1 | 616.3 | 645.2 |

**Table S6. Genetic offset values for each population across climate scenarios.** Genetic offset is shown for each population under six climate scenarios combining two emission pathways (SSP2-4.5; SSP5-8.5) and three future time windows (2041-2060, 2061-2080, 2081-2100). Mt. Lemmon emerges as the most vulnerable, with offset values substantially higher than the other three populations across all scenarios. The three remaining populations show similar vulnerability levels with notably lower offset values, suggesting they are better adapted to withstand projected climate changes.
